# Endoplasmic reticulum stress as a mechanistic link between nutrition and inflammatory bowel disease

**DOI:** 10.3389/fnut.2026.1915419

**Published:** 2026-09-11

**Authors:** Merve Seylan, Sule Arslan, Halit Tanju Besler

**Affiliations:** 1 Department of Nutrition and Dietetics, Istanbul Aydin University, Istanbul, Türkiye; 2 Department of Nutrition and Dietetics, Istanbul Nişantaşi University, Istanbul, Türkiye

**Keywords:** endoplasmic reticulum stress, inflammation, inflammatory bowel disease, nutrition, unfolded protein response

## Abstract

Inflammatory bowel diseases (IBD) are chronic inflammatory disorders that arise from the complex interplay among genetic predisposition, immune dysregulation, environmental factors, and disruption of intestinal epithelial barrier integrity. Increasing evidence suggests that endoplasmic reticulum (ER) stress may represent an important mechanistic pathway in this multifactorial pathogenesis. Experimental evidence indicates that dietary patterns and nutrient-derived factors may modulate ER stress responses. In this review, the role of ER stress in IBD is discussed from a mechanistic perspective, with particular emphasis on the effects of nutrition on ER stress pathways and the relationship between these interactions and IBD pathogenesis. The available findings are evaluated according to whether they derive from direct human IBD studies, experimental colitis models, intestinal cellular systems, or indirect non-intestinal models. Evidence derived predominantly from experimental and non-intestinal metabolic models indicates that high-fat and high-fructose exposures can activate PERK- and IRE1-associated signaling and CHOP-related terminal responses; however, their direct effects on intestinal UPR signaling and epithelial barrier integrity in human IBD have not been established. In contrast, polyunsaturated fatty acids, flavonoids, polyphenols, and certain micronutrients have been reported to modulate selected UPR markers and support cellular adaptation in experimental systems, although differences in dose, bioavailability, and study model limit clinical extrapolation. Collectively, current evidence suggests that ER stress may represent an important mechanistic pathway linking nutrition and intestinal inflammation, although direct causal evidence linking nutritional exposures to mucosal ER stress and clinical outcomes in humans remains limited. Controlled dietary studies incorporating standardized, pathway-specific mucosal biomarkers are required before this mechanistic framework can inform personalized nutritional strategies or ER-stress-targeted interventions.

## Graphical Abstract

**Figure:**
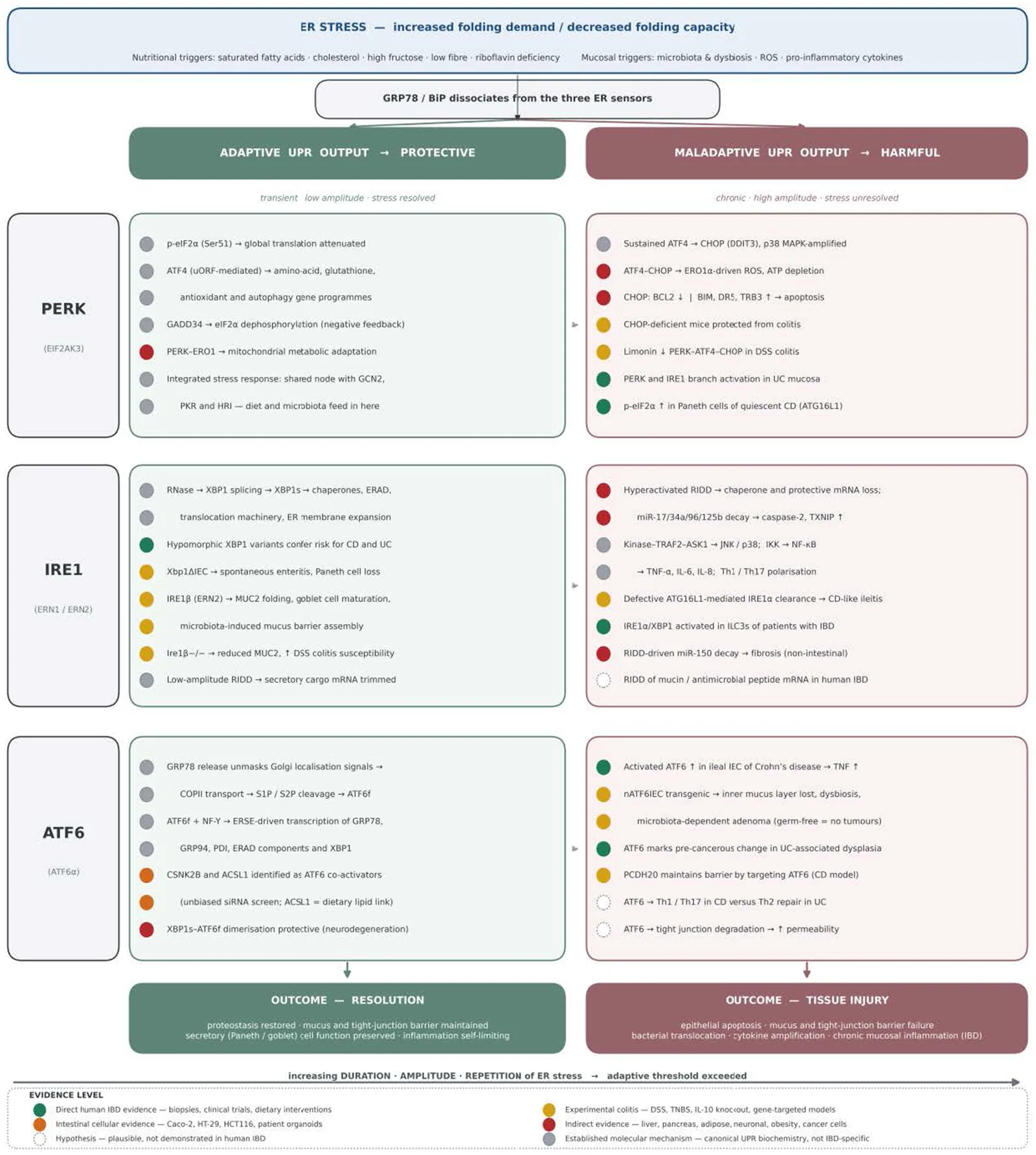

## 1 Introduction

Inflammatory bowel diseases (IBD), primarily including Crohn's disease and ulcerative colitis, represent a group of chronic, lifelong inflammatory disorders that significantly impair patients' quality of life. The pathogenesis and progression of these diseases are shaped by a complex interplay between genetic predisposition, immune responses, environmental exposures, and disruptions in the integrity of the intestinal epithelial barrier (1). In recent years, direct human IBD evidence derived from clinical and epidemiological studies has increasingly supported the involvement of endoplasmic reticulum (ER) stress as a key cellular mechanism contributing to this multifactorial pathogenesis (2, 3).

At the molecular level, it is well established that ER stress arises when the protein-folding capacity of the cell is exceeded, triggering an adaptive mechanism known as the unfolded protein response (UPR), which aims to restore cellular homeostasis. However, when this response becomes chronic, protective mechanisms are replaced by apoptosis, inflammation, and tissue damage. Evidence from experimental colitis models indicates that, characterized by a high protein synthesis capacity—persistent activation of ER stress contributes to the disruption of mucosal barrier integrity and the amplification of inflammatory responses (4).

Nutrition is one of the environmental factors capable of directly modulating ER stress. Dietary macro- and micronutrients can influence ER function at multiple levels, including membrane composition, protein folding processes, oxidative capacity, and inflammatory signaling. Diets rich in saturated fats and simple carbohydrates, as well as specific fatty acids, polyphenols, and micronutrients, have been shown to modulate ER stress responses. The relationships between nutrition and ER stress are supported predominantly by indirect evidence, as summarized within the stratified evidence base presented in Table 1. These findings suggest that nutrition may serve not only as a tool for symptom management but also to influence the underlying pathophysiological processes of IBD.

**Table 1:** Effects of nutritional and nutraceutical exposures on ER stress pathways, stratified by level of evidence.

References	Model	Tissue	Nutritional exposure	Dose	Duration	UPR pathway	Molecular marker	Inflammation outcome	Barrier outcome	Evidence level	Limitation
Direct human IBD evidence — patient biopsies, clinical trials, dietary interventions											
Hanai et al. (130)	Randomized, double-blind, placebo-controlled trial (*n* = 89)	Quiescent UC — clinical and endoscopic	Curcumin + sulfasalazine/mesalamine	2 g/day (1 g twice daily)	6 months	Not assessed	None measured	Relapse 4.65% vs. 20.51% with placebo	Not assessed	Direct human IBD evidence (RCT)	No ER stress endpoint; mechanism entirely inferred; single-country cohort
Lang et al. (131)	Randomized, double-blind, placebo-controlled trial (*n* = 50)	Mild-to-moderate active UC — mucosa	Curcumin added to mesalamine	3 g/day	4 weeks	Not assessed	None measured	Clinical remission 53.8% vs. 0%	Endoscopic remission 38% vs. 0%	Direct human IBD evidence (RCT)	No ER stress endpoint; small sample; short duration
Samsami-Kor et al. (132)	Randomized, double-blind, placebo-controlled pilot trial (*n* = 50)	Active mild-to-moderate UC — plasma and PBMC	Resveratrol	500 mg/day	6 weeks	Not assessed	NF-κB activity in PBMC	↓ TNF-α, ↓ hs-CRP; improved disease activity	Not assessed	Direct human IBD evidence (pilot RCT)	No mucosal sampling; no ER stress endpoint; pilot size
von Martels et al. (119)	Prospective, open-label intervention (*n* = 70)	Crohn's disease — plasma, feces	Riboflavin	100 mg/day (=70 × RDA)	3 weeks	Not assessed	None measured	↓ symptoms; mixed anti-inflammatory effects	Not assessed	Direct human IBD evidence (uncontrolled)	No placebo arm; no mucosal ER stress endpoint; pharmacological dose
Feagan et al. (113)	Two randomized, double-blind, placebo-controlled trials (EPIC-1, EPIC-2)	Crohn's disease in remission — clinical	Omega-3 free fatty acids	4 g/day	52 and 58 weeks	Not assessed	None measured	No significant reduction in relapse vs. placebo	Not assessed	Direct human IBD evidence (large RCT — negative)	Negative result; no mechanistic endpoint; contradicts preclinical data
Chen et al. (133)	Multicentre, double-blind, randomized controlled study (*n* = 1,100)	Colorectal adenoma — colonic mucosa	Berberine	0.3 g twice daily	24 months	Not assessed	None measured	Adenoma recurrence 36% vs. 47%	Not assessed	Direct human evidence — non-IBD population	Adenoma, not IBD; no ER stress endpoint; no dietary source of the agent
Experimental colitis evidence — DSS, TNBS, IL-10 knockout and gene-targeted colitis models											
Song et al. (26)	Mouse, DSS-induced chronic colitis	Colon	Limonin	Not extracted — verify against primary source	Chronic DSS protocol	PERK	ATF4, CHOP; NF-κB	↓ colonic inflammation	↓ epithelial damage	Experimental colitis (DSS)	Rodent only; human equivalent dose not calculated; single compound
Namba et al. (12)	CHOP-deficient mice, experimental colitis	Colon	None (genetic)	—	—	PERK	CHOP	CHOP deficiency protects from colitis	Not assessed	Experimental colitis (genetic)	No nutritional exposure; establishes causality for CHOP only
Guo et al. (121)	High-fat-diet-induced obese mice	Ileum — Paneth cells	Rutin; rutin + inulin	Not extracted — verify against primary source	Not extracted — verify against primary source	Not extracted — verify against primary source	ER stress markers in Paneth cells	↓ inflammatory status	Improved dysbiosis	Experimental — diet-induced obesity model in intestinal tissue	Obesity model, not colitis; doses not extracted
Desai et al. (137)	Gnotobiotic mice colonized with defined human microbiota	Colon — mucus layer	Fiber deprivation	Fiber-free diet	Chronic and intermittent exposure	Not assessed	Muc2; mucus layer thickness	↑ susceptibility to Citrobacter rodentium	Erosion of the colonic mucus barrier	Experimental (gnotobiotic)	UPR not measured; not a colitis model *per se*
Furusawa et al. (136); Kelly et al. (135)	Mouse colitis models; germ-free and gnotobiotic mice	Colon	Butyrate / short-chain fatty acids	Physiological luminal range	Not extracted — verify against primary source	Not assessed	Foxp3; HIF-1α	↓ colitis severity; ↑ colonic Treg	↑ epithelial barrier function	Experimental colitis	UPR endpoints not measured; link to proteostasis is inferential
Intestinal cellular evidence — Caco-2, HT-29, HCT116 and patient-derived organoids											
Ben Salem et al. (128)	HCT116 human colorectal carcinoma cells	Intestinal epithelial cell line	Quercetin (against α- and β-zearalenol)	Not extracted — verify against primary source	24 h (typical)	All three branches	GRP78, CHOP, caspase activation	↓ apoptotic signaling	Not assessed	Intestinal cellular evidence (carcinoma line)	Transformed line, not primary epithelium; toxin model; single concentration
No published study	Caco-2, HT-29 or patient-derived intestinal organoids	Intestinal epithelium	Any of the dietary components reviewed here	—	—	—	—	—	—	DATA GAP	No study has exposed intestinal epithelial cells or organoids to the dietary exposures reviewed here with a UPR readout
Indirect evidence — liver, pancreas, adipose tissue, neuronal and obesity models, cancer cell lines											
Guo et al. (104)	Mouse 3T3-L1 and rat primary preadipocytes	Adipose	Palmitate	0.25–0.75 mM (albumin-complexed)	12–24 h	PERK, IRE1	GRP78, CHOP	↑ inflammatory signaling	Not assessed	Indirect evidence (adipocyte)	Non-intestinal; unbound free fatty acid concentration not reported
Zhang et al. (103)	Primary rat hepatocytes	Liver	α-Linolenic acid against palmitic acid lipotoxicity	0.25–0.5 mM (typical)	24 h	PERK	GRP78, CHOP	Not assessed	Not assessed	Indirect evidence (hepatocyte)	Non-intestinal; no dose–response design
Feng et al. (169)	Mouse peritoneal macrophages	Macrophage	Cholesterol loading	Not extracted — verify against primary source	Not extracted — verify against primary source	All three branches	CHOP	Cholesterol-induced cytotoxicity	Not assessed	Indirect evidence (macrophage)	Non-intestinal; loading model
Savard et al. (102)	Mouse, experimental steatohepatitis	Liver	High fat + dietary cholesterol	Not extracted — verify against primary source	30 weeks (typical)	Not extracted — verify against primary source	CHOP-mediated UPR	Hepatic inflammation	Not assessed	Indirect evidence (liver)	Non-intestinal; doses not extracted
Yuzefovych et al. (99)	Mouse, high-fat-diet-induced insulin resistance	Skeletal muscle, liver	High-fat diet	=60% energy from fat (typical)	16 weeks (typical)	PERK, IRE1	GRP78, CHOP	Not assessed	Not assessed	Indirect evidence (obesity model)	Non-intestinal; dietary composition, not a discrete dose
Zhao et al. (100)	Diet-induced obese rats	Liver	High-fat diet (lard vs. soybean oil)	Not extracted — verify against primary source	Not extracted — verify against primary source	Not extracted — verify against primary source	GRP78, CHOP, eIF2α	Hepatic inflammation	Not assessed	Indirect evidence (liver, obesity model)	Non-intestinal
Yang et al. (106)	Rodent model of insulin resistance	Liver, skeletal muscle	Fish oil	Not extracted — verify against primary source	Not extracted — verify against primary source	AMPK-dependent	GRP78, CHOP, eIF2α	Not assessed	Not assessed	Indirect evidence (metabolic model)	Non-intestinal
Balakumar et al. (109)	Rat	Liver, pancreas	High-fructose diet	Not extracted — verify against primary source	Not extracted — verify against primary source	PERK, IRE1	GRP78, CHOP	Not assessed	Not assessed	Indirect evidence (metabolic model)	Non-intestinal
Baena et al. (110)	Rat	Liver	Fructose supplementation	10% w/v in drinking water (typical)	14 days	None induced	mTORC activation; impaired autophagy	Not assessed	Not assessed	Indirect evidence (liver) — negative for ER stress	Reports NO ER stress induction at short exposure; important negative control for the fructose argument
Stanhope et al. (112)	Human, controlled feeding study	Visceral adipose, liver (metabolic endpoints)	Fructose-sweetened beverages	25% of energy requirement	10 weeks	Not assessed	None measured	Not assessed	Not assessed	Indirect evidence — human, non-intestinal	No intestinal tissue and no UPR endpoint; healthy overweight adults, not IBD
Yan et al. (125)	HT22 mouse hippocampal neuronal cells	Neuronal	Resveratrol	Not extracted — verify against primary source	Not extracted — verify against primary source	Sirt3–autophagy	GRP78, XBP1, CHOP, caspase-12	↓ apoptosis	Not assessed	Indirect evidence (neuronal)	Non-intestinal cell line
Zhang et al. (127)	Osteoblasts; murine osteolysis model	Bone	Quercetin	Not extracted — verify against primary source	Not extracted — verify against primary source	PERK, IRE1	GRP78, PERK, IRE1, CHOP	↓ inflammatory signaling	Not assessed	Indirect evidence (bone)	Non-intestinal
Manthey et al. (116); Xin et al. (118)	HepG2 human hepatoma cells	Liver	Riboflavin deficiency	Riboflavin-depleted medium	Not extracted — verify against primary source	Not extracted — verify against primary source	Ero1/PDI system; CHOP	Not assessed	Not assessed	Indirect evidence (hepatoma line)	Non-intestinal; deficiency model rather than dietary intake
Brito et al. (review) (107)	Various obesity and metabolic models	Liver, adipose tissue	Apigenin, berberine, caffeic acid, curcumin, hydroxytyrosol	Not extracted — verify against primary source	Not extracted — verify against primary source	Variable	GRP78, CHOP, eIF2α, ATF4	↓ inflammatory markers	Not assessed	Indirect evidence — secondary source	Data derived from a review of obesity models; primary doses not independently verified; no intestinal data

Rows are grouped as direct human IBD evidence, experimental colitis evidence, intestinal cellular evidence and indirect evidence, according to the classification defined in the Introduction. “Not extracted” indicates that the parameter is not reported in the form required for this table and should be verified against the primary source before submission; “Not assessed” indicates that the outcome was not measured in the cited study. The distribution of rows is itself informative: the mechanistic basis for the nutrition–ER stress relationship rests almost entirely on indirect, non-intestinal models, whereas the human evidence consists of clinical trials that did not measure ER stress. CD, Crohn's disease; PBMC, peripheral blood mononuclear cell; RCT, randomized controlled trial; RDA, recommended daily allowance; UC, ulcerative colitis; UPR, unfolded protein response. ↑ indicates an increase or upregulation, whereas ↓ indicates a decrease or downregulation.

The aim of this review is to examine the relationship between ER stress, nutrition, and inflammatory bowel diseases from a mechanistic perspective, to elucidate the potential role of ER stress in IBD pathogenesis, and to discuss how nutrition may act as a regulatory or triggering factor within this axis. This approach is expected to deepen current understanding of the role of nutrition in IBD and to provide a conceptual framework for future research.

**Figure 1:**
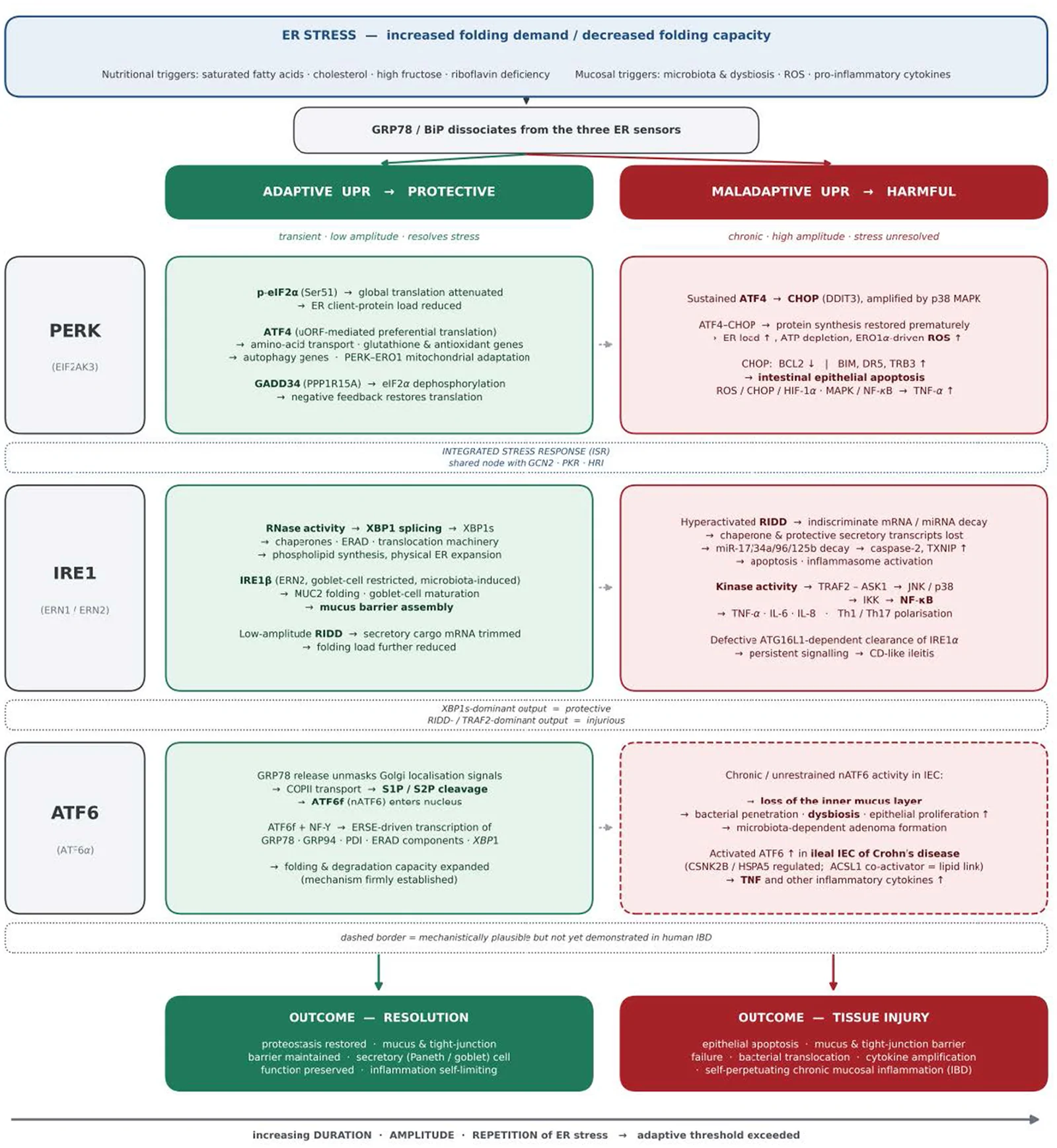
Adaptive vs. maladaptive outputs of the three UPR branches in the intestinal epithelium. Nutritional and mucosal triggers increase the ER folding burden and promote the dissociation of GRP78/BiP from PERK, IRE1, and ATF6. The left side of the figure illustrates adaptive or protective outputs, whereas the right side illustrates maladaptive or potentially harmful outputs. The transition between these responses is influenced by the duration, amplitude, and repetition of ER stress. The figure integrates established molecular mechanisms with evidence derived from human IBD, experimental colitis, and intestinal cellular models, while highlighting areas in which the proposed mechanisms remain hypothetical. ERAD, ER-associated degradation; IEC, intestinal epithelial cell; RIDD, regulated IRE1-dependent decay; ROS, reactive oxygen species.

Because the strength of the evidence varies greatly between the claims made in this field, and because that variation is frequently obscured when mechanistic and clinical findings are cited side by side, the present review applies an explicit evidence classification throughout. Four categories are used. Direct human IBD evidence comprises patient biopsies and surgical specimens, clinical trials and dietary intervention studies conducted in patients with inflammatory bowel disease. Experimental colitis evidence comprises chemically induced models (dextran sulfate sodium, trinitrobenzene sulfonic acid), spontaneous models such as the interleukin-10 knockout mouse, and gene-targeted colitis models. Intestinal cellular evidence comprises studies in intestinal epithelial cell lines (Caco-2, HT-29, HCT116) and in patient-derived intestinal organoids and enteroids. Indirect evidence comprises studies conducted in non-intestinal systems—hepatocytes, pancreas, adipose tissue, neuronal and other cell lines, models of obesity and insulin resistance, and cancer cell lines—from which conclusions about the intestine can only be extrapolated. Statements that are mechanistically plausible but have not been demonstrated in any of these settings are identified as hypotheses. Each paragraph of the sections that follow closes with the evidence level on which it rests, each table carries an evidence-level column, and Figure 1 applies the same classification as a color code. Canonical UPR biochemistry established in non-disease-specific systems is identified separately, since it underpins the entire field but is not in itself evidence about IBD. This methodological framework defines the evidence classification applied throughout the review.

### 1.1 Literature search and evidence base

This article is a narrative review. The literature underpinning it was identified through a structured search of three bibliographic databases—PubMed (MEDLINE), Scopus and the Web of Science Core Collection—using a three-block Boolean strategy that combined the molecular exposure, the disease context and the nutritional determinants of interest:

(“endoplasmic reticulum stress” OR “ER stress”) AND (IBD OR Crohn OR “ulcerative colitis”) AND (diet OR nutrition OR microbiota OR polyphenol)

The strategy was executed in the TITLE-ABS-KEY field in Scopus and in the Topic field in the Web of Science, and without field restriction in PubMed; no limits were applied on publication year, document type or study design. The search returned 492 records (PubMed 114, Scopus 161, Web of Science 217), which reduced to 294 unique records after deduplication on Digital Object Identifier, PubMed identifier and normalized title. Only 26.2% of these records were indexed in all three databases and 33.7% were unique to the Web of Science, confirming that no single database would have provided adequate coverage of this literature.

The deduplicated set was screened against the themes developed here—UPR sensor signaling, genetic susceptibility, autophagy crosstalk, dietary and microbial modulation of epithelial proteostasis, and clinical translation. Records addressing ER stress outside an intestinal context, non-research document types and publications without an English full text were not considered further. The reference list additionally includes foundational endoplasmic reticulum stress literature from outside the intestinal field, which lies beyond the scope of the search strategy but is required to support the mechanistic argument developed here. Because this review is interpretive rather than quantitative, no formal risk-of-bias assessment and no quantitative synthesis were undertaken; instead, the strength of the evidence supporting each claim is stated explicitly in the text and in the accompanying tables.

## 2 ER stress as a mechanistic link in IBD

Endoplasmic reticulum stress is a fundamental cellular stress response that arises from the accumulation of misfolded or unfolded proteins within the cell, thereby threatening cellular homeostasis. This stress response can be triggered by various factors, including toxin exposure, increased oxidative stress, disruptions in energy metabolism, inflammation, and pharmacological agents. Under these conditions, the unfolded protein response is activated to restore protein-folding capacity and re-establish cellular equilibrium. The UPR is regulated through three principal signaling pathways: inositol-requiring enzyme 1 (IRE1), protein kinase RNA-like ER kinase (PERK), and activating transcription factor 6 (ATF6). Under physiological conditions, these sensors remain inactive through their association with chaperone proteins such as GRP78. Upon the onset of ER stress, GRP78 dissociates from these sensors to bind misfolded proteins, leading to their activation and the initiation of downstream cellular responses (4, 5). Collectively, these events describe an established molecular mechanism in canonical UPR biochemistry; however, they do not constitute IBD-specific evidence (6–8).

Accumulating experimental and clinical evidence over the past years indicates that ER stress plays a significant role in the development and progression of inflammatory bowel diseases. Elevated levels of ER stress markers have been consistently reported in animal models as well as in cellular, organoid, and tissue samples derived from patients with IBD (2, 9). Notably, in individuals with Crohn's disease, reduced expression of the chaperone protein GRP78 in the intestinal mucosa has been observed, accompanied by increased levels of the transcription factor C/EBP homologous protein (CHOP) and an altered XBP1s/XBP1u ratio. These findings suggest a sustained and dysregulated ER stress response (2, 3, 9). Accordingly, these observations integrate direct human IBD evidence from patient-derived intestinal tissue and intestinal cellular evidence from cellular and organoid samples, while experimental studies further support a mechanistic contribution of disrupted UPR signaling and ER stress to intestinal inflammation and colitis (10, 11).

When ER stress becomes chronic, cytoprotective mechanisms are progressively impaired, giving way to apoptosis and tissue damage. Increased production of reactive oxygen species via CHOP-mediated pathways accelerates epithelial cell death, leading to disruption of mucosal integrity and exacerbation of inflammation (12). This process contributes to transmural tissue damage and fistula formation in Crohn's disease, as well as to persistent mucosal inflammation in ulcerative colitis (13). Collectively, ER stress has moved from being regarded as a secondary consequence of inflammation to being considered one of the mechanistic pathways that may link diet and intestinal inflammation, although the evidence supporting each step of that link differs in strength. Accordingly, the proposed mechanistic sequence is supported primarily by experimental colitis and indirect evidence, whereas the available human data remain correlative.

A concept that is essential for interpreting the sections that follow is that the UPR is not a unidirectional stress signal but a temporally biphasic programme. During the early, low-amplitude phase, all three sensors operate in an adaptive (cytoprotective) mode: translational load is transiently reduced, chaperone and ER-associated degradation (ERAD) capacity is expanded, and the ER membrane itself is remodeled, so that folding demand and folding supply are re-matched and the cell survives (14, 15). When the insult is intense, repetitive or chronic—as is the case in the inflamed intestinal mucosa—the same sensors switch to a maladaptive (terminal) mode in which apoptotic and pro-inflammatory transcriptional programmes dominate over reparative ones. Whether a given UPR branch is protective or pathogenic in IBD therefore cannot be answered in the abstract; the answer depends on the duration and amplitude of the signal, the cell type in which it occurs, and which downstream effector is engaged. This adaptive-vs.-maladaptive dichotomy is summarized in Figure 1 and is used as the organizing framework throughout Sections 3.1–3.3. This framework synthesis established canonical UPR biology and does not itself constitute primary IBD-specific evidence.

### 2.1 PERK signaling: the integrated stress response and the adaptive-to-terminal switch

PERK (protein kinase RNA-like ER kinase, EIF2AK3) is a type I ER-resident transmembrane protein whose luminal domain is held inactive by GRP78/BiP. Accumulation of unfolded polypeptides titrates GRP78 away from PERK, allowing the sensor to oligomerise and trans-autophosphorylate. The activated cytosolic kinase domain has one principal substrate: serine 51 of the α-subunit of eukaryotic translation initiation factor 2 (eIF2α). Phosphorylated eIF2α (p-eIF2α) converts the eIF2 complex from a substrate into a competitive inhibitor of its own guanine nucleotide exchange factor, eIF2B, which lowers the availability of the eIF2–GTP–Met-tRNAi ternary complex and thereby attenuates cap-dependent translation initiation globally (14, 15). The immediate consequence is a reduction in the flux of nascent polypeptides entering the ER lumen—the fastest available means of decompressing a saturated folding machinery. This signaling sequence represents an established molecular mechanism of canonical UPR biochemistry and does not itself constitute IBD-specific evidence (16).

PERK is not, however, an exclusively ER-dedicated kinase. It is one of four mammalian eIF2α kinases—alongside GCN2 (amino acid deprivation), PKR (double-stranded RNA and viral infection) and HRI (heme deficiency, oxidative and mitochondrial stress)—that converge on the identical Ser51 residue. This convergence defines the integrated stress response (ISR), an established molecular signaling hub in which nutritional, microbial, oxidative and proteostatic inputs are read out through a single common node (17, 18). The ISR framework is directly pertinent to the theme of the present review, because indirect evidence suggests that dietary amino acid limitation, micronutrient deficiency and lipotoxic or oxidative stress can all raise p-eIF2α in intestinal epithelial cells (IECs) without necessarily producing classical ER protein misfolding. Interpreting elevated p-eIF2α or ATF4 in intestinal tissue as evidence of ER stress alone is therefore an oversimplification, and studies in this field increasingly require ISR-specific controls before the signal can be attributed to PERK rather than to a sister kinase.

Paradoxically, the same translational block that silences most transcripts permits the preferential translation of a small set of mRNAs containing inhibitory upstream open reading frames (uORFs), the best characterized of which encodes activating transcription factor 4 (ATF4). When ternary complex availability is low, scanning ribosomes bypass the inhibitory uORF and initiate instead at the ATF4 coding sequence, so that ATF4 protein rises while bulk synthesis falls (19). ATF4 is the principal effector of the adaptive arm of this branch: it induces amino acid transporters and aminoacyl-tRNA synthetases, glutathione biosynthesis and other antioxidant genes, autophagy-related genes and—critically—GADD34 (PPP1R15A), the regulatory subunit that recruits protein phosphatase 1 to dephosphorylate eIF2α. GADD34 thereby closes a negative feedback loop that restores translation once folding capacity has been re-established (17). Together, these events constitute an established molecular mechanism within adaptive PERK signaling. In the intestinal epithelium, PERK–p-eIF2α-dependent autophagy provides a compensatory mechanism that partially buffers secretory cells against proteostatic overload, indirect evidence further suggests that PERK additionally interacts with the oxidoreductase ERO1 to couple ER redox status to mitochondrial metabolic adaptation (20). Within this window, PERK activity is unequivocally cytoprotective.

If the insult is not resolved, the temporal profile of ATF4 activity changes and the branch convert to a terminal programme. Sustained ATF4 induces CCAAT/enhancer-binding protein homologous protein (CHOP/DDIT3), whose transcriptional activity is further amplified by p38 MAPK-mediated phosphorylation (21). ATF4 and CHOP then act as a functional pair that, counterintuitively, restores rather than suppresses protein synthesis—chiefly by inducing GADD34 to excess and by driving transcription of genes encoding secretory proteins—which increases ER client load, ATP consumption and, through ERO1α-driven hyperoxidation of the ER lumen, reactive oxygen species (ROS) production (22). In parallel, CHOP represses the anti-apoptotic BCL2 while inducing pro-apoptotic BIM, death receptor 5 (DR5) and TRB3, tipping the mitochondrial apoptotic balance (5). The distinction between the adaptive and the terminal phase is therefore not a change of pathway but a change of dose and duration within the same pathway: the PERK branch first buys the cell time and then, if that time is not used successfully, executes it. Although this adaptive-to-terminal switch is an established molecular mechanism, its relevance to intestinal inflammation and IBD is supported here by indirect evidence from non-intestinal cell culture systems and animal models (23).

Evidence supporting the relevance of this switch to IBD comes from several complementary levels. Direct human IBD evidence shows that colonic mucosa from patients with ulcerative colitis shows activation of both the PERK and the IRE1 branches relative to controls (24), and that ER stress markers are broadly upregulated in IBD tissue in association with TLR2-dependent inflammatory signaling (25). Experimental colitis evidence further demonstrates that CHOP is not a bystander: animals deficient in CHOP are protected from experimental colitis, which establishes a causal, disease-promoting role for the terminal effector of this axis rather than a merely correlative one (12). Pharmacological interruption of the axis is likewise protective, since limonin attenuates dextran sulfate sodium-induced chronic colitis specifically by inhibiting PERK–ATF4–CHOP signaling and downstream NF-κB activation (26). At the interface with inflammation, CHOP acts upstream of cytokine production, with ROS/CHOP/HIF-1α and MAPK/NF-κB pathways jointly driving TNF-α expression (27), while CHOP-dependent ROS generation accelerates epithelial cell death and mucosal barrier failure (5, 28).

Two implications follow for the nutrition–ER stress–IBD axis. First, because dietary stimuli feed into the ISR at the level of eIF2α phosphorylation, nutritional exposures can shift the position of the adaptive/terminal set point rather than simply switching the pathway on or off. Second, therapeutic strategies directed at this branch cannot be directionally naive: global PERK inhibition would remove the protective translational brake together with the pro-apoptotic output, whereas selective suppression of the CHOP arm, or pharmacological restoration of ternary complex availability downstream of p-eIF2α, would preserve adaptation while limiting terminal signaling. These implications are interpretive rather than conclusions drawn directly from primary IBD data. To date such approaches have been tested only in preclinical models, and no PERK- or ISR-targeted agent has been evaluated in human IBD.

### 2.2 IRE1 signaling: dual enzymatic activities, isoform specificity, and inflammatory output

Inositol-requiring enzyme 1 (IRE1) is the most evolutionarily conserved of the three sensors and the only one that possesses two distinct catalytic activities within a single polypeptide: a cytosolic serine/threonine kinase domain and a carboxy-terminal endoribonuclease (RNase) domain. Activation requires both the release of GRP78 and the direct binding of unfolded polypeptides to the luminal domain, which promotes dimerization and higher-order oligomerization; trans-autophosphorylation by the kinase domain then induces the conformational rearrangement that licenses the RNase domain (14). This functional separation is not a technical detail. As set out below, the kinase output and the RNase output have largely opposite consequences for the intestinal epithelium, so that the same molecule may be protective or pathogenic depending on which of its two enzymatic functions predominates. The activation sequence described above constitutes an established molecular mechanism of canonical UPR biochemistry, whereas the intestinal consequences of the two outputs are evaluated in the following sections according to their respective evidence levels.

Mammals express two IRE1 paralogues. IRE1α (ERN1) is ubiquitously expressed and mediates the canonical responses described below. IRE1β (ERN2) has a highly restricted expression pattern confined to the mucosal epithelium of the gastrointestinal and respiratory tracts, and within the intestine it is enriched in goblet cells. The two isoforms are not redundant. IRE1β is a comparatively weak XBP1 splicer but a potent RNase, and it can attenuate IRE1α signaling. Functionally, experimental colitis evidence shows that IRE1β-deficient mice have reduced intestinal MUC2 production and markedly increased susceptibility to dextran sulfate sodium-induced colitis (29). More recently, evidence from germ-free models showed that ERN2/IRE1β is required for microbiota-induced goblet cell maturation and assembly of the colonic mucus barrier: ERN2 expression is induced only after colonization of the alimentary tract by normal microflora, and its deletion reduces MUC2-positive goblet cell numbers and causes accumulation of misfolded MUC2 precursors in colonic secretory progenitors—a phenotype not reproduced by intestine-specific ERN1 deletion, despite the ability of IRE1α to splice Xbp1 (30). Limited human data, restricted to colorectal cancer expression analyses, indicate that IRE1β expression is downregulated in colorectal cancer (31). IRE1β therefore constitutes an epithelium-specific, mucus-protective node through which the microbiota can act upon barrier integrity; by extension, these findings support the hypothesis that diet-driven changes in microbial composition may influence the barrier through the same pathway (32).

The best characterized RNase output of IRE1α is the unconventional cytoplasmic splicing of X-box binding protein 1 (XBP1) mRNA. The RNase excises a 26-nucleotide intron and the exon halves are re-ligated by RtcB, producing a translational frameshift that yields XBP1s, a potent basic leucine zipper transcription factor. XBP1s expands folding capacity by inducing chaperones, ERAD components, protein translocation machinery and phospholipid biosynthesis for physical expansion of the ER (14). This is the canonical adaptive output of the branch, and the intestinal epithelium is unusually dependent upon it. Experimental colitis evidence demonstrates that epithelium-specific deletion of Xbp1 in mice produces spontaneous small intestinal inflammation with crypt abscesses, leukocyte infiltration and ulceration, driven by Paneth cell loss, goblet cell abnormality and an epithelium that is hyper-responsive to flagellin and TNF-α (33). The same study also provided direct human IBD evidence through the identification of hypomorphic XBP1 variants as genetic risk factors for both Crohn's disease and ulcerative colitis. Together, these findings indicate that loss of the adaptive IRE1–XBP1 output is sufficient to initiate IBD-like disease in experimental models and that XBP1 variation is genetically associated with human IBD, providing evidence that is considerably stronger than that available for the other two branches (34).

The RNase domain has a second substrate repertoire. In addition to XBP1, activated IRE1α cleaves ER-localized mRNAs, selected microRNAs and transfer RNAs bearing XBP1-like stem-loop consensus sites, a process termed regulated IRE1-dependent decay (RIDD) (35). At low signal amplitude, RIDD is adaptive: degrading mRNAs encoding secretory cargo reduces the incoming folding load and complements the XBP1s programme. When IRE1α signaling is sustained or hyperactivated, however, RIDD becomes indiscriminate and destroys transcripts encoding chaperones, ER-resident and protective secretory proteins, and anti-apoptotic microRNAs—including miR-17, miR-34a, miR-96, and miR-125b—thereby de-repressing caspase-2 and TXNIP and promoting apoptosis and inflammasome activation (36). RIDD amplitude thus constitutes a molecular timer that converts IRE1α from a pro-survival into a pro-death effector. This amplitude-dependent RIDD switch represents an established molecular mechanism. In the intestinal epithelium, indirect evidence suggests that IRE1α and IRE1β protect the mucosa through distinct mechanisms, and their combined loss results in reduced expression of defense-associated transcripts alongside increased expression of inflammatory and pathogenic ones. It should be emphasized, however, that the more specific proposition that RIDD-mediated degradation of mucin and antimicrobial peptide transcripts contributes to barrier failure in human IBD remains a mechanistically plausible hypothesis rather than a demonstrated finding, since RIDD substrates have not yet been cataloged in patient-derived intestinal tissue.

Independently of its RNase function, the phosphorylated kinase domain of IRE1α serves as a signaling scaffold. It recruits TNF receptor-associated factor 2 (TRAF2), which engages apoptosis signal-regulating kinase 1 (ASK1) to activate JNK and p38 MAPK, and which also engages the IκB kinase complex to liberate NF-Kb (37). The downstream consequence is transcription of TNF-α, IL-6 and IL-8, so that a proteostatic signal is converted directly into a pro-inflammatory one without any requirement for XBP1. This IRE1α-TRAF2 cascade constitutes an established molecular mechanism linking proteostatic stress to inflammatory signaling. Experimental colitis evidence indicates that this kinase-dependent output amplifies mucosal inflammation, favors T helper 1 and T helper 17 polarization and drives epithelial apoptosis (25), whereas available human tissue data are consistent with activation of the same axis but remain correlative (9). Failure to terminate the signal is itself pathogenic: ATG16L1-dependent selective autophagy normally clears activated IRE1α from the ER, and when this removal is defective IRE1α accumulates and drives Crohn's disease-like ileitis (38). Consistent with this, compound deficiency of XBP1 and ATG16L-1 in the epithelium produces transmural, Crohn's-like ileitis originating from Paneth cells (39), and NOD2, autophagy and ER stress converge upon a shared pathogenic axis in Crohn's disease (40).

The adaptive and the pro-inflammatory faces of IRE1 can therefore be assigned to specific molecular outputs rather than to the sensor as a whole. An XBP1s-dominant output, together with IRE1β-dependent maintenance of goblet cell function, is protective; a RIDD-dominant or TRAF2–ASK1–JNK/NF-κB-dominant output is injurious. This assignment represents an interpretive synthesis of the evidence presented above rather than a conclusion derived from direct comparative testing. The distinction has direct therapeutic relevance, since kinase-inhibiting RNase attenuators and allosteric RNase-site inhibitors act upon different outputs of the same protein and would be expected to have opposite effects on the adaptive arm. All such agents remain at the preclinical stage in intestinal inflammation.

### 2.3 ATF6 signaling: established evidence, open hypotheses, and IBD-specific data

The molecular activation mechanism of ATF6 is firmly established. ATF6α is a type II ER transmembrane protein containing a cytosolic basic leucine zipper domain (41). Under basal conditions, GRP78/BiP binding masks two Golgi localization signals within its luminal domain; ER stress-induced dissociation of GRP78 unmasks these signals and permits COPII-dependent trafficking to the Golgi apparatus (42). There, ATF6 is cleaved sequentially by site-1 and site-2 proteases, releasing the cytosolic fragment ATF6f (also termed nATF6), which enters the nucleus and, together with NF-Y, binds ER stress response elements to induce chaperones (including GRP78 itself), ERAD components and XBP1 transcription. These steps are supported by direct biochemical and structural evidence and may be regarded as settled.

Evidence specific to the intestinal epithelium is more recent but now includes direct human data. In an unbiased RNA interference screen covering 7,783 genes and using an ATF6-dependent ER stress response element reporter, casein kinase 2 beta (CSNK2B) and acyl-CoA synthetase long-chain family member 1 (ACSL1) were identified and validated as upstream co-activators of ATF6α signaling. Critically for the present review, direct human IBD evidence showed that ileal IECs from patients with Crohn's disease contained higher levels of activated ATF6 regulated by CSNK2B and HSPA5 (43). Supporting intestinal cellular evidence from functional experiments in these cells and in organoids derived from Atg16l1ΔIEC and Xbp1ΔIEC mice showed that ATF6 increased the expression of TNF and other inflammatory cytokines in response to ER stress (43). Together, these findings support a pro-inflammatory role for ATF6 in the IBD epithelium, but causality is established by the cellular models rather than by the human tissue observations alone. The identification of ACSL1, an enzyme of long-chain fatty acid activation, is of additional nutritional relevance and supports the hypothesis that dietary lipid supply could modulate the gain of ATF6 signaling in IECs through this candidate molecular route. A second line of epithelium-specific experimental evidence comes from transgenic mice expressing activated nATF6 selectively in the intestinal epithelium. These animals develop spontaneous colonic adenomas by 12 weeks of age, preceded by loss of the mucus barrier, bacterial penetration into the inner mucus layer, altered caecal microbiota and increased epithelial proliferation; antibiotic treatment markedly reduces and germ-free housing completely prevents tumorigenesis, while elevated ATF6 expression is associated with shorter disease-free survival in patients with colorectal cancer (44). Two caveats are important when this model is invoked in an IBD context. First, it is a model of microbiota-dependent tumorigenesis rather than of colitis, and overt inflammation was not observed at either the pre-tumor or the tumor stage. Second, it employs constitutive expression of the already-cleaved, active fragment, and therefore models chronic maximal ATF6 output rather than physiological ATF6 activation. The model demonstrates that unrestrained ATF6 activity is sufficient to disrupt the mucus barrier and reshape the microbiota—findings of clear relevance to IBD—but it does not establish that ATF6 drives inflammation in IBD itself (45–47).

Direct human evidence from ulcerative colitis-associated dysplasia specimens is observational and correlative. ATF6 immunoreactivity is detectable in colorectal cancers but not in normal colonic mucosa, increases with the degree of cellular atypia, and is present in pre-cancerous atypical lesions in both sporadic and ulcerative colitis-associated colorectal cancer, where ATF6 immunohistochemistry distinguishes low-grade dysplasia from regenerative inflammatory epithelium more accurately than p53 (48). These findings support ATF6 as a candidate biomarker of neoplastic progression in long-standing colitis, but they do not address its role during the inflammatory phase of the disease.

Several propositions concerning ATF6 in IBD that are frequently repeated should, in our view, be presented explicitly as hypotheses rather than as established facts. The proposal that ATF6 preferentially promotes Th1 and Th17 responses in Crohn's disease while supporting Th2-associated mucosal repair in ulcerative colitis derives largely from pattern-level inference and from studies conducted in non-intestinal systems; the evidence cited here does not include an assessment of ATF6-dependent T helper polarization in an IEC-specific loss-of-function intestinal model. Similarly, the suggestion that chronic ATF6 signaling promotes degradation or dephosphorylation of tight junction proteins and thereby raises intestinal permeability rests on association rather than on direct demonstration of ATF6-dependent tight junction turnover in intestinal tissue; the mucus barrier defect in nATF6-transgenic mice is well documented, whereas a tight junction-specific mechanism is not. By contrast, a direct direct Crohn's disease-specific mechanistic link is the demonstration that protocadherin 20 maintains intestinal barrier function by targeting ATF6 (49), while the hypothesis that combined XBP1s and ATF6f activity is protective derives from models of neurodegeneration rather than from intestinal ones (50).

Three gaps define the present limits of knowledge with direct evidence lacking for each of the following questions. No common ATF6 variant has emerged as an IBD susceptibility locus in the genome-wide association studies reviewed here, in contrast to XBP1, ORMDL3, and AGR2, so a primary genetic contribution of this branch remains unsupported. The function of the ATF6β paralogue in the intestine is essentially unstudied. An intestinal epithelium-specific Atf6 loss-of-function colitis model was not identified in the literature reviewed here, which means that the necessity—as opposed to the sufficiency—of ATF6 for intestinal inflammation remains formally untested. ATF6 is accordingly best described at present as a branch with demonstrated pro-inflammatory and barrier-disruptive capacity in the intestinal epithelium, but whose net directional contribution to human IBD remains to be resolved. Adaptive vs. Maladaptive UPR Output as an Organizing Framework.

Taken together, Sections 2.1–2.3 indicate that the three UPR branches cannot be classified as intrinsically protective or intrinsically harmful. Each of them carries an adaptive output that restores proteostasis and preserves the epithelial barrier and a maladaptive output that promotes epithelial apoptosis, barrier failure and cytokine amplification; the variable that determines which output dominates is the duration, amplitude, and repetition of the ER stress signal, together with the cell type in which it occurs. Figure 1 presents this framework schematically, aligning each branch against its adaptive (protective) and maladaptive (harmful) effector arms, and indicating with a broken outline those elements for which the mechanism is plausible but has not yet been demonstrated in human IBD. This framework is an interpretive synthesis of the preceding evidence rather than a conclusion derived directly from primary data. Framed in this way, the nutritional modulators discussed in Section 5 are best understood not as agents that switch ER stress on or off, but as factors that displace the threshold at which the adaptive response is exhausted and the maladaptive response takes over.

### 2.4 Genetic susceptibility and ER stress in IBD

The preceding sections described how the three UPR branches behave under stress. A separate and equally important question is why the intestinal epithelium of some individuals reaches the maladaptive threshold more readily than that of others. Direct human genetic evidence from genome-wide association studies and, more recently, monogenic and rare-variant analyses have converged on an unexpected observation: a substantial proportion of IBD susceptibility loci encode proteins involved in secretory protein folding, ER-associated degradation, autophagy, or microbial sensing rather than in immune effector function as such (51). These genetic findings suggest that ER stress in IBD may not be solely an acquired consequence of inflammation and support the hypothesis that, in genetically predisposed individuals a constitutively narrowed margin of proteostatic reserve may exist before any environmental exposure occurs. This interpretation matters for the argument of the present review, because it implies that nutritional stressors may act upon a substrate whose adaptive capacity is already variable between individuals (52–54).

XBP1 provides the paradigm. As described in Section 2.2, direct human IBD evidence identified hypomorphic XBP1 variants as risk factors for both Crohn's disease and ulcerative colitis, the same study that demonstrating spontaneous enteritis following epithelium-specific Xbp1 deletion (34). The importance of this finding lies in its direction: the risk allele reduces adaptive UPR output rather than increasing stress signaling. Genetic susceptibility in this axis is therefore best conceptualized as a deficit of adaptation, which reframes the therapeutic question from how to suppress the UPR to how to support it.

A second locus, AGR2, encodes an ER-resident protein disulfide isomerase of the secretory epithelium. AGR2 is required for the formation of the disulfide bonds that permit MUC2 to oligomerise correctly, and experimental colitis evidence shows that Agr2-deficient mice fail to produce normal intestinal mucus, develop terminal ileitis and colitis, and exhibit goblet cell depletion with Paneth cell abnormality and constitutive UPR activation (55). Direct human IBD evidence subsequently established the relevance of this pathway to human disease: biallelic loss-of-function AGR2 variants cause a monogenic form of infantile-onset inflammatory bowel disease with mucus barrier dysfunction (56). Additional experimental colitis evidence links AGR2-dependent proteostasis to microbiota, as Agr2-associated ER stress promotes expansion of adherent-invasive *Escherichia coli* and triggers CD103-positive dendritic cell, IL-23-dependent ileocolitis (57). AGR2 thus illustrates the complete chain from a folding-specific gene defect to barrier failure, dysbiosis, and adaptive immune activation.

ORMDL3, one of the most replicated IBD and asthma susceptibility loci, encodes an ER membrane protein that regulates sphingolipid synthesis and ER calcium homeostasis; and indirect mechanistic evidence indicates that its overexpression alters SERCA-dependent calcium handling and modulates the cellular stress response, including the ATF6 branch (58). More recent indirect evidence from human macrophage studies has connected ORMDL3 to the ER–mitochondria interface: by showing that it maintains ER–mitochondria contact sites and thereby regulates NLRP3 inflammasome activation (59). Direct human IBD evidence from a cohort of patients with ulcerative colitis showed that higher ORMDL3 expression was associated with greater disease activity and elevated IL-1β whereas experimental colitis evidence demonstrated that Ormdl3 knockdown reduced the severity of experimental colitis (59). ORMDL3 therefore places a proteostasis-adjacent risk gene directly within innate immune effector signaling. It should be noted, however, that the causal variant at this locus and the direction of its effect in the intestinal epithelium remain incompletely defined.

ER-associated degradation constitutes a distinct arm of proteostasis that has been comparatively neglected in the IBD literature. Terminally misfolded ER proteins are retrotranslocated and degraded by the proteasome, and the SEL1L–HRD1 complex represents the most conserved branch of this machinery. Experimental colitis evidence shows that epithelium-specific deletion of Sel1L in mice causes spontaneous enteritis, profound Paneth cell secretory defects, increased susceptibility to pathogen-induced ileitis and to experimental colitis, and expansion of the mucolytic organism Ruminococcus gnavus (60). Limited direct human IBD evidence shows reduced expression of SEL1L and HRD1 in ileal tissue from patients with Crohn's disease although this observation remains correlative (60). In experimental models, ERAD failure therefore produces a phenotype closely resembling that of defective UPR signaling, which is consistent with the view that total proteostatic capacity, rather than any single sensor, that determines epithelial resilience.

Microbial sensing intersects this network at more than one level. NOD1 and NOD2 are cytosolic pattern recognition receptors whose variants are among the strongest genetic risk factors for Crohn's disease, and they have conventionally been discussed in the context of bacterial peptidoglycan recognition and autophagy. Indirect evidence from bacterial infection models and cell culture provides a more direct mechanistic connection: NOD1 and NOD2, acting through RIP2, are required for ER stress-induced IL-6 production and NF-κB activation, so that the UPR itself functions as a signal that these receptors detect and transduce into inflammation (61). Within these experimental systems, this finding reframes NOD2 as a node at which proteostatic and microbial signals are integrated rather than as a purely microbial sensor, and it supports the hypothesis that a diet- or microbiota-induced increase in epithelial ER stress could be converted into cytokine output in NOD2 risk allele carriers. Together, these indirect findings support the convergence of NOD2, autophagy and ER stress (40), although the studies cited here provide no direct human IBD evidence for the ER stress–NOD component of this axis (62).

ATG16L1 links the genetic and the autophagy arms of this network and is discussed further in Section 3.1. Experimental colitis evidence shows that mice carrying hypomorphic Atg16l1 display striking Paneth cell abnormalities that phenocopy those observed in patients homozygous for the ATG16L1 T300A risk allele (63). In these models, the phenotype is conditional upon an environmental trigger, since it requires concurrent norovirus infection and is further modified by commensal bacteria and by injury (64). Critically for the present argument, direct human IBD evidence from genotype-stratified biopsies shows that patients with quiescent Crohn's disease who carry the ATG16L1 risk allele have high Paneth cell expression of GRP78 and phosphorylated eIF2α even in the absence of active inflammation, and this Paneth cell ER stress correlates with bacterial persistence (65). These findings provide strong evidence that genotype-associated Paneth cell ER stress can be present without concurrent active inflammation; however, because human observations are cross-sectional, they do not by themselves establish that ER stress preceded disease onset.

Mitochondrial stress represents the final component of this network and is increasingly recognized as inseparable from ER stress, both because the two organelles are physically coupled at mitochondria-associated membranes and because oxidative protein folding is bioenergetically expensive. Experimental colitis evidence from mice with intestinal epithelium-specific loss of the mitochondrial chaperone HSP60 shows that this perturbation activates the mitochondrial unfolded protein response and that mitochondrial function controls epithelial stemness and proliferation (66). Further experimental evidence indicates that mitochondrial impairment drives the transition of intestinal stem cells into dysfunctional Paneth cells, whereas direct human IBD evidence from a postoperative Crohn's disease cohort shows that the corresponding mitochondrial–Paneth cell phenotype predicts disease recurrence (67). The nutritional regulation of PERK signaling and ER oxidative protein-folding capacity through the ERO1/PDI system has been discussed in Section 4.1, where the effects of dietary fatty acids, carbohydrates, and riboflavin on ER proteostasis were examined.

Two features emerging from an interpretive synthesis of this network deserve emphasis. First, its components converge anatomically upon the secretory lineages of the small intestinal epithelium: XBP1, AGR2, SEL1L, ATG16L1, and NOD2 all produce Paneth cell or goblet cell phenotypes, which is consistent with the exceptional proteostatic burden carried by professional secretory cells. Second, several of these genetic lesions interact with environmental inputs—including microbial colonization, infection, injury, or diet—for the expression of a disease phenotype. This gene–environment structure is consistent with ER stress serving as a point of convergence and provides amechanistic rationale for the hypothesis that nutritional interventions may have genotype-dependent effects. Experimental colitis evidence provides proof of principle that this axis is pharmacologically tractable since chemical chaperones reduce protein misfolding and attenuate colitis in mice (68), however, the studies cited here include no equivalent human intervention stratified by proteostasis genotype.

### 2.5 ER stress and autophagy crosstalk

Autophagy is the principal mechanism by which cells dispose of protein aggregates and damaged organelles that exceed the capacity of the proteasome, and its activation as a cell-survival response following ER stress constitutes an established molecular mechanism (69). Experimental colitis evidence indicates that the two systems are functionally interdependent in the intestinal epithelium, and the genetic architecture described above—in which ATG16L1, NOD2, and XBP1 produce overlapping Paneth cell phenotypes—illustrates this interdependence.

Established molecular evidence derived primarily from non-intestinal cell culture shows that the UPR engages the autophagy machinery through each of its branches, with specific effectors assigned to defined nodes. The PERK arm acts transcriptionally: ATF4 induces ATG3, BECN1 (Beclin-1), MAP1LC3B (LC3), ATG12 and ATG16L1, whereas the ATF4–CHOP heterodimer induces ATG5, ATG10 and GABARAP, and additionally drives expression of ATG7 and of the selective autophagy cargo receptors SQSTM1/p62 and NBR1 (70). This means that the same PERK arm that executes terminal apoptosis when stress is unresolved is also the arm that builds the autophagic apparatus during the adaptive phase—a further illustration of the dose- and duration-dependence emphasized in Section 2.1. The IRE1 arm acts post-translationally: kinase-dependent recruitment of TRAF2 and ASK1 activates JNK, and JNK1-mediated phosphorylation of BCL2 releases Beclin-1 from the inhibitory BCL2–Beclin-1 complex, thereby permitting assembly of the Beclin-1–VPS34 initiation complex (71, 72). XBP1s contributes transcriptionally to BECN1 expression in parallel. Nutrient and energy status enters through a third route, since mTORC1 suppresses and AMPK activates autophagy by opposing phosphorylation of ULK1 (73); this represents a direct molecular junction at which caloric load, amino acid availability, and the cellular AMP/ATP ratio are integrated with proteostatic demand. The cited evidence, however, does not directly confirm these mechanisms in the intestinal epithelium.

Induction of autophagy is not, however, equivalent to effective degradation. Autophagic flux—the rate at which cargo is delivered to and degraded within lysosomes—depends additionally on autophagosome–lysosome fusion and on lysosomal biogenesis, the latter being coordinated by the transcription factor TFEB, which is itself released from mTORC1-dependent inhibition under nutrient deprivation and lysosomal stress (74). This distinction carries a methodological consequence that applies to the interpretation of studies cited throughout this review and is frequently overlooked in nutritional literature. Increased LC3-II or accumulated p62 at a single time point cannot discriminate between enhanced autophagosome formation and blocked lysosomal clearance; the two have opposite biological meanings, and flux must be measured with lysosomal inhibitors or tandem fluorescent reporters. Reports that a dietary compound “induces autophagy” on the basis of static LC3-II measurement alone should therefore be interpreted cautiously, and this caveat applies to several of the polyphenol studies discussed in Section 5.

In IBD the pathological significance of this crosstalk is well documented in both directions. Loss of autophagic capacity aggravates ER stress: experimental colitis evidence shows that hypomorphic Atg16l1 mice develop Paneth cell secretory abnormalities (63), whereas direct human IBD evidence links the human T300A risk allele to elevated GRP78 and phosphorylated eIF2α in Paneth cells (65). Additional experimental colitis evidence shows that compound epithelial deficiency of Xbp1 and Atg16l1 generates transmural, Crohn's disease-like ileitis that neither lesion produces alone (39). Conversely, experimental colitis evidence demonstrates that autophagy is required to terminate UPR signaling: ATG16L1-dependent selective autophagy removes activated IRE1α from the ER, and failure of this clearance causes IRE1α to accumulate and drive Crohn's disease-like ileitis (38). Autophagy in this setting is thus not merely a downstream consequence of ER stress but part of the mechanism that resolves it, which explains why defects in either system produce a convergent phenotype.

The nutritional implications are based primarily on indirect evidence from cell culture and rodent models. Within these experimental settings, several of the dietary compounds discussed in Section 5 act at identifiable nodes of this network: caffeic acid modulates ATF4, CHOP, and eIF2α while inducing ATG5 and ATG7, placing it on the PERK–ATF4 transcriptional arm, and resveratrol reduces ER stress and apoptosis markers while enhancing autophagy and antioxidant defense. Because AMPK and mTORC1 constitute the point at which energy and amino acid status are read, these findings support the hypothesis that interventions altering caloric load or feeding pattern may influence autophagic capacity independently of any specific bioactive compound. It should be stated plainly, however, that this hypothesis has not been tested in human intestinal tissue, and that the available intestinal data derives almost entirely from rodent models and cell lines.

Finally, because no clinical intervention data are available, the therapeutic logic proposed here remains interpretive and mirrors that developed for the UPR branches themselves. Autophagy is generally cytoprotective in the intestinal epithelium under conditions of cellular stress, although excessive or dysregulated autophagic activity may contribute to cell dysfunction or death in other settings. The available direct human IBD evidence remains observational, with abnormal activation of autophagy-associated crinophagy described in Paneth cells of patients with Crohn's disease (75). Strategies targeting this axis should therefore be framed as restoring appropriate autophagic flux where clearance is impaired, rather than as indiscriminately maximizing autophagy.

The preceding sections have treated the intestinal epithelium as a single compartment. This simplification is untenable at the mechanistic level, because the proteostatic burden carried by an epithelial cell is a direct function of its secretory output, and that output differs substantially across the lineages of the crypt–villus axis. A professional secretory cell discharging granules of densely disulfide-bonded protein operates near the ceiling of its folding capacity under physiological conditions, whereas an absorptive enterocyte does not. It follows that an identical systemic stressor—a dietary lipid load, a microbial shift, a cytokine exposure—will not produce an identical UPR in every epithelial cell, and that the phenotype of any given genetic or nutritional perturbation is largely determined by which lineage is first pushed past its adaptive threshold. This section examines the lineages individually.

Paneth cells occupy the extreme end of this spectrum and are correspondingly the most frequently affected lineage in the models discussed above. Their granules contain lysozyme, α-defensins and other antimicrobial peptides whose maturation depends on extensive oxidative folding. Experimental colitis evidence from gene-targeted mouse models shows that epithelium-specific deletion of Xbp1 causes Paneth cell loss (34); hypomorphic Atg16l1 produces Paneth cell granule abnormalities that phenocopy those of human ATG16L1 T300A homozygotes (63); Sel1L deletion abolishes Paneth cell secretory function (60); and combined Xbp1 and Atg16l1 deficiency generates transmural, Crohn's disease-like ileitis originating from this cell type (39). Complementary direct human IBD evidence comes from genotype-stratified biopsies and a postoperative Crohn's disease cohort: patients with quiescent Crohn's disease who carry the ATG16L1 risk allele show elevated Paneth cell GRP78 and phosphorylated eIF2α in the absence of active inflammation (65), and mitochondrial impairment drives intestinal stem cells into a dysfunctional Paneth cell state that predicts postoperative recurrence (67). The convergence of five genetically distinct lesions upon one cell type is not coincidental; taken together, these findings support the interpretation that the Paneth cell is a proteostatic bottleneck of the ileal epithelium and, by extension, the hypothesis that Paneth cell dysfunction may represent a cellular origin of ileal Crohn's disease (76, 77).

Goblet cells present a comparable but mechanistically distinct vulnerability. MUC2 is among the largest proteins produced by any mammalian cell and requires ordered disulfide-dependent oligomerization before secretion, a process that depends on the ER-resident protein disulfide isomerase AGR2 (55). Direct human IBD evidence for this pathway comes from biallelic AGR2 loss-of-function variants that cause infantile-onset IBD (56). Evidence from experimental colitis and germ-free mouse models identifies two UPR outputs support this lineage specifically. The goblet cell-restricted sensor IRE1β is induced by microbial colonization and is required for goblet cell maturation and inner mucus layer assembly (29, 30), and XBP1 is required for MUC2 expression and mucus layer formation, with epithelial Xbp1 deficiency markedly aggravating necroptosis-induced colitis by permitting bacterial contact with the epithelial surface (78). Goblet cell UPR failure therefore translates into barrier failure through a mucus-specific route rather than through the antimicrobial-peptide route that predominates in Paneth cells—a distinction consistent with the colonic and mucosal distribution of ulcerative colitis, although, as noted in Section 6, this correspondence remains a hypothesis rather than a demonstrated disease-subtype mechanism (79).

Enterocytes carry a lower constitutive folding burden but are the lineage in which nutrition and the UPR intersect most directly, because dietary lipid must be assembled into chylomicrons within the ER. The goblet-associated sensor IRE1β also acts in this compartment: it degrades the mRNA encoding microsomal triglyceride transfer protein through RIDD, thereby limiting chylomicron output, and Ire1β-deficient mice fed high-cholesterol, high-fat diets develop more pronounced hyperlipidaemia with increased intestinal—but not hepatic—MTP expression (80). Although this evidence is limited to a dietary mouse model and no corresponding human data is currently available, the observation is mechanistically important for the present review. It demonstrates that a UPR sensor functions constitutively in the intestine as a regulator of dietary lipid handling rather than only as an emergency stress transducer, and it identifies RIDD as the specific output through which it does so. It also supports the hypothesis that the enterocyte response to a high-fat diet is not merely passive lipotoxic injury but an active, sensor-mediated metabolic adjustment whose failure may contribute to the effects of Western dietary patterns described in Section 5.

Intestinal stem cells exhibit the inverse relationship, and the direction of this effect is frequently misunderstood. Markers of ER stress and UPR activity correlate inversely with markers of stemness: stem cells maintain low basal UPR activity, whereas UPR activation is a feature of transit-amplifying and differentiated cells. Experimentally induced ER stress causes rapid loss of stemness, driving Lgr5-positive cells out of the stem compartment and into differentiation (81). Conversely, hypomorphic Xbp1 function instructs a multilayered regenerative response, with IRE1α-mediated expansion of Lgr5- and Olfm4-positive stem cells and a Stat3-dependent increase in transit-amplifying cell output, and Xbp1 loss also promotes intestinal tumorigenesis (82). Taken together, experimental colitis and other animal models, supported by intestinal cellular evidence, indicate that the UPR operates in this lineage as a differentiation switch rather than only as a survival mechanism. In the absence of direct human IBD evidence, its proposed disease-level consequences remain hypotheses: chronic mucosal ER stress may deplete the regenerative reserve required for mucosal healing, and interventions that alter epithelial ER stress may have effects on proliferation and neoplastic risk that are distinct from their effects on inflammation. Two lineages must currently be classified as data gaps. Tuft cells, which initiate type 2 immunity through IL-25 release, and enteroendocrine cells, which package and secrete peptide hormones and therefore might reasonably be expected to carry a substantial ER load, have not to our knowledge been examined for UPR activity or UPR dependence in either experimental colitis or human IBD. No cell type-specific UPR reporter or conditional deletion study has been reported for either lineage. This absence is worth stating explicitly rather than inferring a mechanism by analogy with Paneth and goblet cells, and it is taken up among the research priorities identified in the final section.

### 2.6 ER stress in immune and stromal cells

ER stress in IBD is not confined to the epithelium. Several immune lineages activate the UPR constitutively as part of normal differentiation, which means that in these cells the UPR is not a stress response at all but a developmental programme; in others it is engaged by inflammatory cytokines and pattern recognition receptor signaling. Distinguishing these two situations is essential, because a therapeutic agent directed at a UPR sensor would act simultaneously upon epithelial adaptation and upon immune effector function, in some cases in opposite directions (51, 83).

Macrophages provide the clearest example of non-canonical UPR engagement. Indirect evidence from macrophage cell culture shows that ligation of TLR2 and TLR4 activates IRE1α and XBP1 splicing in the absence of any ER stress, and TLR-activated XBP1 is required for optimal and sustained production of pro-inflammatory cytokines (84). This finding carries a specific implication for the argument developed in Section 2.2. In the intestinal epithelium XBP1s is the adaptive, protective output; in the macrophage the same transcription factor is a pro-inflammatory effector. The direction of XBP1 function is therefore cell-type dependent, and statements about XBP1 in IBD are meaningful only when the compartment is specified. The same logic applies to ORMDL3, which sustains NLRP3 inflammasome activation in cultured human macrophages by maintaining ER–mitochondria contacts, whereas direct human IBD evidence from an ulcerative colitis cohort is correlative: ORMDL3 expression tracks with disease activity and IL-1β in ulcerative colitis (59).

Indirect evidence from animal and cell-culture models indicates that dendritic cells likewise maintain constitutive IRE1α and XBP1 activity in the steady state. Loss of XBP1 in CD11c-positive cells impairs ER homeostasis, phenotype, and antigen presentation by CD8α-positive conventional dendritic cells (85). Dendritic cell fate is additionally governed by the second RNase output: RIDD sets the threshold for dendritic cell survival, so that the balance between XBP1 splicing and RIDD determines whether the cell persists (86). The RIDD-vs.-XBP1s dichotomy introduced in Section 2.2 for the epithelium therefore recurs in the antigen-presenting compartment, supporting the interpretation that these outputs may constitute distinct pharmacological targets rather than as a single “IRE1 pathway.” This proposition, however, has not been examined in intestinal or IBD-specific settings.

Plasma cells represent the developmental extreme of UPR utilization. Indirect evidence from animal and cell-culture studies indicates that XBP1 is required for plasma cell differentiation (87), and it governs the late events of that differentiation—expansion of the secretory apparatus and of immunoglobulin output—while being dispensable for antigen-specific memory B cell development (88). The intestinal lamina propria contains the largest plasma cell population in the body, and IgA output in IBD is substantial. In the absence of IBD-specific data, any intervention that suppresses XBP1 output systemically would therefore be predicted, rather than demonstrated, to compromise mucosal humoral immunity, a consideration absent from most discussions of UPR-targeted therapy.

Indirect evidence from animal and cell-culture models indicates that regulatory T cells are destabilized rather than supported by ER stress. Inflammatory cytokines induce an ER stress response that suppresses FoxP3 expression, and deletion of the ERAD E3 ligase Hrd1 elevates ER stress-responsive gene expression in Tregs and markedly diminishes their suppressive function under inflammatory conditions (89). ERAD capacity, discussed in Section 4 as an epithelial determinant, therefore also governs the stability of the principal tolerogenic lymphocyte population, providing a second and immunological route by which proteostatic failure could favor chronic intestinal inflammation.

Innate lymphoid cells have only recently been examined in this context, but direct human IBD evidence is now available and is supported by experimental colitis models. Intestinal group 3 innate lymphoid cells display a circadian rhythm of IRE1α/XBP1 expression, and this pathway is activated in ILC3s from mice with experimental colitis and in sorted ILC3s isolated from inflamed tissue of patients with IBD, where pathway activation sustains cytokine output (90). ILC3s thus join macrophages and dendritic cells as a compartment in which IRE1α/XBP1 signaling is pro-inflammatory, in contrast to its protective role in the epithelium (91, 92).

Stromal cells contribute to different clinical problems. Fibroblast activation and collagen deposition underlie stricture formation in Crohn's disease, whereas indirect evidence from non-intestinal fibroblast systems and liver and skin fibrosis models indicates that ER stress promotes fibrosis through IRE1α-mediated degradation of miR-150 together with XBP1 splicing (93). Because RIDD-mediated microRNA degradation is one operative mechanism, the miR-150-degradation arm represents a further instance in which the injurious output of IRE1α is RNase-dependent but XBP1-independent. Intestinal fibrosis is not addressed elsewhere in this review, and in the absence of intestinal stricture data, the proposed link between epithelial or stromal ER stress and human stricturing disease remains an indirect extrapolation from these non-intestinal systems.

Two additional compartments must currently be classified as data gaps rather than summarized mechanistically. Neutrophils are abundant in active IBD and contribute to mucosal injury through reactive oxygen species and extracellular trap formation (94), and endothelial dysfunction with disruption of the gut vascular barrier is increasingly recognized as a component of IBD pathogenesis (95); yet neither compartment has been characterized for UPR branch activity in intestinal tissue, and no conditional deletion of a UPR sensor has been reported in either lineage in a colitis model. Together with the tuft and enteroendocrine lineages noted above, these represent defined and addressable gaps, and they are listed among the priorities in the final section rather than filled by extrapolation here.

The following two conclusions are interpretive syntheses of the evidence surveyed above rather than claims derived from new primary data. First, the functional direction of a given UPR output is compartment dependent: XBP1s is protective in the epithelium and pro-inflammatory in macrophages, dendritic cells and ILC3s, while ERAD capacity supports both epithelial secretion and Treg stability. Any therapeutic strategy directed at the UPR must therefore specify its cellular target, and systemic sensor inhibition should be expected to produce opposing effects in different compartments simultaneously. Second, because nutritional exposures reach all these compartments—directly through circulating lipids and micronutrients, and indirectly through microbiota—the net effect of a dietary intervention on mucosal inflammation cannot be predicted from epithelial data alone. This consideration frames the nutritional evidence presented in the following section.

## 3 Nutritional modulation of ER stress pathways

Before individual nutrients are considered, two methodological points must be made explicit, because they determine how much weight the evidence in this section can bear. The first concerns dose. Much of the mechanistic literature on nutritional modulation of ER stress derives from cell culture, in which fatty acids and polyphenols are applied at concentrations that may exceed those attainable *in vivo*, and from rodent studies in which the administered dose expressed per kilogram of body weight cannot be transferred directly to humans; allometric conversion based on body surface area rather than body weight is required for any meaningful estimate of a human equivalent dose (96, 97). *In vitro* fatty acid experiments carry an additional requirement, since it is the unbound free fatty acid concentration rather than the nominal one that determines lipotoxicity, and this depends on the albumin-to-fatty-acid ratio, which is frequently not reported (98). The second point concerns study design: a compound tested at a single concentration provides no information about the threshold at which its effect appears, and a human equivalent dose calculated from such a study is therefore uninformative. Table 2 accordingly summarizes dose, exposure duration, experimental model and the estimated human equivalent for the principal agents discussed below, and states explicitly where no conversion is possible rather than supplying a figure that the underlying study cannot support.

**Table 2:** Dose, exposure duration, experimental model, and estimated human equivalent for the principal nutritional and nutraceutical modulators of ER stress discussed in this review.

Agent	Dose and duration	Model	Estimated human equivalent	Reported endpoint	Evidence level
Fructose	25% of energy requirement, 10 weeks	Human, controlled feeding	Not applicable (human study); within upper habitual intake	↑ visceral adiposity, DNL, postprandial triglycerides; ↓ insulin sensitivity; intestinal UPR not measured	Indirect evidence — human, non-intestinal
High-fat diet	45%−60% energy from fat, 8–16 weeks	Rodent	Not convertible — dietary composition, not a discrete dose	↑ GRP78, CHOP, IRE1; mitochondrial dysfunction	Indirect evidence — rodent metabolic models
Palmitic acid (SFA)	0.25–0.5 mM, 6–24 h (typical)	Cell culture, albumin-complexed	Not calculable — unbound FFA concentration rarely reported	↑ PERK, CHOP; apoptosis	Indirect evidence — non-intestinal cell culture
α-Linolenic acid, fish oil, olive oil	Single concentrations; no dose–response design	Cell culture and rodent	Not calculable	↓ GRP78, CHOP, p-eIF2α; attenuation of palmitate lipotoxicity	Indirect evidence — cell culture and rodent metabolic models
Omega-3 free fatty acids	4 g/day, 52 weeks (EPIC-1) and 58 weeks (EPIC-2)	Human RCT, CD maintenance	Not applicable (human study)	No significant reduction in relapse vs. placebo	Direct human IBD evidence — large RCT (negative)
Riboflavin	100 mg/day, 3 weeks	Human, open-label, CD (*n* = 70)	Not applicable; =70 × RDA — pharmacological	↓ systemic oxidative stress and symptoms; ↑ fecal butyrate; mucosal UPR not measured	Direct human IBD evidence (uncontrolled) + indirect (HepG2)
Curcumin	2 g/day, 6 months (maintenance); 3 g/day, 4 weeks (induction)	Human RCT, UC	=65–100 g turmeric/day — not attainable through diet	↓ relapse; ↑ remission when added to mesalamine	Direct human IBD evidence (RCT); mechanism indirect only
Resveratrol	500 mg/day, 6 weeks	Human RCT, UC (*n* = 50)	≫ habitual dietary intake (single-digit mg) — not attainable through diet	↓ TNF-α, hs-CRP, NF-κB activity in PBMCs	Direct human IBD evidence (pilot RCT); mechanism indirect only
Quercetin	10–100 μM (typical); no dose–response design	Cell culture	Not calculable	↓ GRP78, PERK, IRE1, CHOP; ↓ cytokine production	Intestinal cellular evidence (HCT116) + indirect (bone)
Berberine	0.3 g twice daily, 24 months	Human RCT (*n* = 1,100)	No dietary source — pharmacological agent	Adenoma recurrence 36% vs. 47% with placebo	Direct human evidence — non-IBD population
Hydroxytyrosol	5 mg/day (regulatory claim threshold)	Human, regulatory assessment	=20 g olive oil/day — dietarily attainable	Protection of blood lipids from oxidative damage; intestinal ER stress not assessed	Indirect evidence — regulatory assessment, non-intestinal
Caffeic acid	Single concentration reported	Cell culture	Not calculable	Modulation of ATF4, CHOP, eIF2α; ↑ ATG5, ATG7	Indirect evidence — secondary source
Butyrate	10–20 mM luminal (physiological range)	Human and mouse colonic lumen	Determined by fiber intake rather than by supplement dose	HIF stabilization, barrier reinforcement, Treg induction; UPR endpoints untested	Experimental colitis; UPR endpoints untested

Human equivalent doses, where calculable, are based on body surface area normalization (95, 96). “Not calculable” indicates that the underlying study used a single concentration or dose without a dose–response design, so that no threshold can be inferred. CD, Crohn's disease; DNL, de novo lipogenesis; FFA, free fatty acid; RCT, randomized controlled trial; RDA, recommended daily allowance; UC, ulcerative colitis.

### 3.1 Dietary components

Dietary patterns and nutrient intake play a critical role in maintaining endoplasmic reticulum function. Excessive energy intake and imbalanced macronutrient distribution can overwhelm the protein-folding capacity of the ER, thereby inducing ER stress. The supporting evidence is indirect and derives primarily from rodent obesity and insulin-resistance models involving liver, skeletal muscle, and adipose tissue. In these non-intestinal models, high-fat diet (HFD) exposure progressively increases ER stress, leading to mitochondrial dysfunction, oxidative stress, and the activation of autophagy and protein degradation pathways (99). Comparable intestinal data are not currently available.

Beyond the quantity of dietary fat, the type of fatty acids represents a key determinant of ER stress severity. The supporting evidence is indirect and derives primarily from rodent feeding studies and non-intestinal hepatocyte, preadipocyte, and macrophage models. Diets rich in saturated fatty acids (SFAs) have been shown to induce higher expression of PERK and CHOP compared to diets enriched in polyunsaturated fatty acids (PUFAs). In contrast, transitioning to a low-fat diet has been associated with attenuation of ER stress and reduced hepatic steatosis (100). Mechanistically, SFAs can alter the phospholipid composition of the ER membrane, impair its function and activate the unfolded protein response even in the absence of protein misfolding. This suggests a direct cytotoxic potential of SFAs (101). Furthermore, high SFA intake has been reported to synergistically enhance ER stress in the presence of cholesterol, with cholesterol accumulation in ER membranes activating CHOP-mediated UPR pathways and promoting apoptosis (102). *In vitro* studies have also shown that palmitic acid induces ER stress and inflammation, whereas PUFAs such as α-linolenic acid can mitigate ER stress-mediated apoptosis induced by lipotoxic fatty acids (103–105). Similarly, unsaturated fat sources such as fish oil and olive oil have been shown to suppress ER stress markers including GRP78, CHOP, and eIF2α in various animal models (106, 107). Specifically, the comparisons of saturated with polyunsaturated fatty acids on PERK and CHOP expression derive from rodent feeding studies, and the palmitate findings from cell culture using albumin-complexed fatty acid at concentrations typically in the 0.25–0.5 mM range; whether the unbound free fatty acid concentrations so generated fall within the postprandial physiological range cannot be judged unless the albumin-to-fatty-acid ratio is reported (98). No corresponding intestinal evidence is available from experimental models, and no human study has measured intestinal mucosal ER stress markers in response to a defined saturated fat load (108).

Simple carbohydrates represent another important dietary determinant of ER stress, although the supporting mechanistic evidence is indirect. In rodent hepatic and pancreatic models, high-fructose diets have been shown to activate PERK and IRE1 pathways, like high-fat diets, leading to increased CHOP expression and apoptosis. Notably, combined exposure to high fat and fructose results in the most pronounced increase in ER stress markers and apoptotic responses (109). The ER stress-inducing effects of fructose appear to be time-dependent, as short-term or low-dose exposure may not trigger ER stress, whereas prolonged exposure significantly increases CHOP expression and caspase activation (110, 111). These findings highlight that the impact of nutrition on ER stress is influenced not only by nutrient composition but also by duration of exposure. The closest available human dose–response data concern fructose but are non-intestinal and therefore provide only indirect evidence for the present question. In a controlled feeding study, fructose-sweetened beverages providing 25% of energy requirements for 10 weeks increased visceral adiposity, hepatic *de novo* lipogenesis and postprandial triglycerides and reduced insulin sensitivity, whereas isocaloric glucose did not (112). This intake is high but lies within the upper range of habitual consumption, which distinguishes fructose from most of the compounds discussed later in this section. Intestinal ER stress endpoints were not assessed in that study, and no human study has yet related to fructose intake to mucosal UPR activation.

The evidence concerning polyunsaturated fatty acids requires particular care, because the mechanistic and the clinical literatures point in different directions. The protective effects described above—attenuation of palmitate-induced ER stress by α-linolenic acid, and suppression of GRP78, CHOP and eIF2α phosphorylation by fish oil and olive oil—were obtained in cultured cell lines and in rodent models of diet-induced metabolic stress, and none of these studies was conducted in patients or measured intestinal mucosal endpoints (103–107). This indirect evidence establishes that PUFAs can modulate ER stress signaling under experimental conditions; it does not establish clinical benefit. Direct human IBD evidence regarding clinical efficacy comes from two large randomized, double-blind, placebo-controlled trials that evaluated omega-3 fatty acids for the maintenance of remission in Crohn's disease. With 4 g of omega-3 free fatty acids administered daily for 52 and 58 weeks respectively, relapse rates did not differ significantly from placebo: 31.6% vs. 35.7% in EPIC-1 and 47.8% vs. 48.8% in EPIC-2 (113). The mechanistic plausibility of PUFA-mediated attenuation of ER stress therefore remains, and it is supported by consistent cell and animal data; but in randomized controlled trials in humans, omega-3 supplementation has not prevented relapse in Crohn's disease, and this is the conclusion that must be carried forward (114, 115).

Micronutrients also play an essential role in ER function. Riboflavin is a key cofactor in the Ero1/PDI system, which is required for oxidative protein folding within the ER. Riboflavin deficiency disrupts this system, leading to the accumulation of misfolded proteins and activation of ER stress responses (116, 117). Increased CHOP expression and enhanced apoptosis have been observed under conditions of riboflavin deficiency, highlighting its importance in maintaining ER homeostasis (21, 118). Table 1 shows the effects of nutritional factors on ER stress pathways and their potential implications for IBD. Riboflavin is also the micronutrient for which the most direct human data exists. In the RISE-UP study, 100 mg of riboflavin daily for 3 weeks in 70 patients with Crohn's disease reduced systemic oxidative stress and clinical symptoms, with mixed anti-inflammatory effects (119). This dose is approximately 70 times the recommended daily intake and is therefore a pharmacological rather than a dietary exposure. Separately, a randomized, placebo-controlled study in healthy adults reported that riboflavin supplementation increased fecal butyrate production without gross compositional change in the microbiota (120). Mucosal ER stress markers were not measured in either study, so the link between riboflavin repletion and epithelial UPR activity in patients remains inferential.

### 3.2 Nutraceuticals and pharmacological doses

Importantly, the effects of nutrition on ER stress are not limited to pro-stress stimuli. Indirect evidence from obesity and metabolic models indicates that certain dietary components exert protective effects by attenuating ER stress and enhancing cellular adaptive responses. Flavonoids and polyphenols have been shown to reduce ER stress marker expression, thereby alleviating lipid accumulation and inflammation. For instance, apigenin has been demonstrated to suppress ER stress-related protein expression and reduce lipid accumulation *in vitro* models. Similarly, berberine supplementation has been associated with decreased GRP78 and CHOP levels, along with reduced macrophage infiltration and IL-6 expression (107). Emerging evidence also suggests that some flavonoids exert their effects through modulation of the gut microbiota. For example, indirect intestinal evidence from high-fat-diet-fed obese mice indicates that rutin administration has been shown to reduce ER stress markers in the intestine, potentially through improvements in microbiota composition accompanied by decreased lipopolysaccharide and pro-inflammatory cytokine levels. The combination of rutin and inulin further enhances these protective effects (121–124).

Certain phenolic compounds modulate ER stress through activation of autophagy pathways. For caffeic acid, curcumin, hydroxytyrosol, and resveratrol, the evidence cited here is indirect and derives from neuronal, osteoblast, and hepatic models. Caffeic acid has been shown to regulate ER stress responses via ATF4, CHOP, and eIF2α, while promoting the expression of autophagy-related proteins such as ATG5 and ATG7, thereby supporting protein homeostasis. Curcumin has been reported to attenuate ER stress by inhibiting eIF2α phosphorylation; however, whether this effect is direct or secondary to reductions in oxidative stress remains unclear. Hydroxytyrosol has been shown to suppress ER stress-related proteins and modulate JNK/IRS signaling pathways, although its precise mechanism of action has not yet been fully elucidated (107). Among polyphenols, resveratrol and quercetin are particularly prominent modulators of ER stress. Resveratrol has been shown to reduce ER stress and apoptosis markers, including GRP78, XBP1, CHOP, and caspase-12, while enhancing autophagy and antioxidant defense mechanisms in *in vitro* models (125, 126). Quercetin, on the other hand, has been evaluated in indirect macrophage models and in one intestinal cellular study using HCT116 cells. Across these systems, it suppresses ER stress signaling and inflammatory cytokine production by downregulating GRP78, PERK, IRE1, and CHOP expression in both intestinal epithelial cells and macrophages (127–129).

A distinction that is frequently blurred in this literature must be drawn explicitly: with the exception of hydroxytyrosol, the compounds described in this subsection are not consumed at these doses as part of any diet. The curcumin trials in ulcerative colitis administered 2 g daily for 6 months as maintenance therapy (130) and 3 g daily for 4 weeks as induction therapy alongside mesalamine (131); since turmeric contains approximately 3% curcumin by weight, these intakes correspond to tens of grams of spice per day and are unattainable through food. The resveratrol trial in ulcerative colitis used 500 mg daily for 6 weeks (132), against a habitual dietary intake from grapes and red wine measured in single-digit milligrams. Berberine, tested at 0.3 g twice daily for 2 years and shown to reduce the recurrence of colorectal adenoma from 47% to 36% (133), is a plant alkaloid rather than a nutrient and has no meaningful dietary source. Hydroxytyrosol is the exception: the European Food Safety Authority recognizes a health claim at an intake of 5 mg per day of hydroxytyrosol and its derivatives, obtainable from approximately 20 g of a suitable olive oil (134), which places it within dietary reach. These agents should therefore be evaluated as pharmacological interventions, with the corresponding requirements for dose-finding, pharmacokinetic characterization and safety assessment, and their effects should not be presented as evidence for the benefit of the foods from which they are derived. It should further be noted that none of the human trials cited here measured mucosal ER stress markers; the ulcerative colitis trials therefore provide direct human IBD evidence for clinical endpoints only, whereas the ER stress mechanism attributed to these compounds rests entirely on indirect cell and animal evidence and were not tested in those trials.

### 3.3 Microbiota-mediated modulation of ER stress

The influence of nutrition upon epithelial ER stress is not exerted solely by nutrients acting directly on the enterocyte. A substantial part of it is mediated by the gut microbiota, which converts dietary substrate into metabolites that reach the epithelium at local concentrations far exceeding those of the parent nutrient. Three considerations make this route especially relevant here: the microbial metabolite profile is determined largely by diet; several of these metabolites act upon pathways that intersect directly with the UPR; and, as established in Section 2.2, the epithelium-specific sensor IRE1β is itself induced by microbial colonization, providing a direct mechanistic link between the microbiota and epithelial UPR capacity (30).

Short-chain fatty acids are the principal products of bacterial fermentation of dietary fiber and resistant starch. The barrier and immunoregulatory effects summarized below are supported by experimental colitis and cell-culture evidence. Butyrate is the preferred oxidative substrate of the colonocyte and stabilizes epithelial hypoxia-inducible factor, thereby reinforcing barrier function (135), and it drives the differentiation of colonic regulatory T cells (136). Propionate contributes to hepatic gluconeogenesis and to peripheral Treg induction but is less consistently associated with epithelial barrier effects. The connection to proteostasis is bioenergetic: butyrate oxidation supplies a substantial share of the ATP required for chaperone function and oxidative protein folding, so an inadequate supply of fermentable substrate would be expected to lower the threshold at which the epithelial UPR becomes maladaptive. This inference is mechanistically coherent and is consistent with the barrier and immunoregulatory effects of butyrate described above, but it has not been tested directly by measuring UPR markers in butyrate-depleted colonic epithelium, and it should be read as a hypothesis. Fiber deprivation acts on the barrier by a second and more direct route. Experimental evidence from gnotobiotic mice shows that when fermentable substrate is withdrawn, the microbiota turns to host-secreted mucin glycoproteins as a nutrient source, expanding mucus-degrading taxa, eroding the colonic mucus layer and increasing susceptibility to enteric pathogens (137). This finding joins the dietary and the proteostatic arguments of the present review at a single point, although UPR endpoints were not measured in that model. The resulting proteostatic link should therefore be regarded as a hypothesis. To compensate for accelerated mucus degradation the goblet cell would be expected to increase MUC2 output, and MUC2 synthesis is precisely the process that depends upon AGR2, IRE1β, and XBP1 (Section 4). A low-fiber Western dietary pattern may therefore raise the secretory demand placed upon the cell type whose proteostatic reserve is already the most constrained, which offers a coherent mechanistic account of how fiber intake could modulate mucosal vulnerability in ulcerative colitis.

Bile acids provide a further axis. Secondary bile acids are generated by bacterial 7α-dehydroxylation of host primary bile acids, a capacity confined to a small number of taxa. Direct human IBD evidence from a UC ileal-pouch cohort shows depletion of lithocholic and deoxycholic acid together with loss of the bacteria and genes required for their production, whereas complementary experimental colitis evidence shows that supplementation with secondary bile acids reduces inflammation in three murine colitis models in a partly TGR5-dependent manner (138). Bile acid pool size and composition are strongly diet-responsive, being shifted by both the quantity and the type of dietary fat, which places this axis directly downstream of the dietary lipid exposures discussed above. Microbial tryptophan catabolism constitutes a parallel route: experimental colitis evidence shows that indole derivatives act as aryl hydrocarbon receptor ligands and promote IL-22 production, and a microbiota unable to generate these ligands aggravates colitis, an effect reversed by AhR agonism or by colonization with tryptophan-metabolizing lactobacilli (139). The availability of tryptophan substrate for this pathway is determined by dietary protein quantity and quality. However, neither study measured ER stress or UPR endpoints, so the connection of these metabolite pathways to proteostasis remains indirect (140–142).

Hydrogen sulfide illustrates the dose dependence that characterizes this entire field. It is generated by sulfate-reducing bacteria from dietary sulfur amino acids and inorganic sulfate, and its effects are biphasic. Evidence from experimental colitis and cell-culture studies supports this biphasic model: at low concentrations it is protective, promoting the resolution of colonic inflammation and restoring mucus production and microbiota biofilm architecture (143); at higher concentrations it inhibits colonocyte butyrate oxidation and cytochrome c oxidase, impairing the energy supply upon which oxidative protein folding depends (144). Because luminal sulfide production rises with high intakes of animal protein and of sulfur-containing food additives, these findings support the hypothesis that a Western dietary pattern could compromise epithelial bioenergetics and, in consequence, proteostatic capacity. However, direct measurement of UPR activation under defined luminal sulfide concentrations has not been reported, and no direct human IBD evidence is available for this relationship; the proposed link between luminal sulfide and mucosal UPR activity therefore remains untested. Finally, microbial recognition feeds back into the UPR at the level of the innate immune receptors themselves. Indirect evidence from macrophage cell culture shows that ligation of TLR2 and TLR4 activates IRE1α and XBP1 splicing in macrophages in the absence of any ER stress (84); associative direct human IBD evidence from tissue samples shows that ER stress markers are upregulated in IBD tissue in association with TLR2-dependent signaling (25); and indirect evidence from infection models shows that NOD1 and NOD2 are required for ER stress-induced IL-6 production and NF-κB activation (61). These receptors therefore do not merely detect bacteria— they read the proteostatic state of the cell and convert it into cytokine output, which is the mechanism providing a mechanistically plausible but as yet untested route by which a diet-induced increase in epithelial ER stress could be amplified into mucosal inflammation in carriers of NOD2 risk alleles. Taken together, the microbial arm of the nutrition–ER stress axis operates through at least four separable mechanisms—epithelial energy supply, mucus turnover, metabolite–receptor signaling and innate receptor-mediated transduction of proteostatic state—and the net effect of any dietary change cannot be predicted from any one of them in isolation (145).

## 4 The interplay between ER stress, nutrition, and IBD

Inflammatory bowel diseases are chronic inflammatory disorders that arise from the complex interplay of genetic predisposition, immune dysregulation, environmental factors, and disruption of epithelial barrier integrity. Recent evidence suggests that endoplasmic reticulum stress may represent an important mechanistic pathway within this network. Dietary patterns and nutrient-derived factors represent key environmental modulators capable of both initiating and regulating ER stress responses. Consequently, the nutrition-ER stress-IBD axis has emerged as a critical biological framework for understanding IBD pathogenesis in an integrated manner (146–148).

Direct human IBD evidence from tissue samples, together with experimental colitis evidence, indicates that in the context of IBD, the chronic activation of ER stress is closely linked to the high protein synthesis demand of intestinal epithelial cells and the continuous renewal of the mucosal barrier. The accumulation of misfolded proteins leads to sustained activation of the unfolded protein response, shifting cellular responses from adaptive mechanisms toward apoptosis and inflammation. Increased CHOP expression plays a key role in this transition by promoting reactive oxygen species (ROS) production and epithelial cell death, ultimately compromising mucosal integrity. This disruption facilitates the translocation of luminal antigens and microbial products, thereby amplifying chronic inflammatory responses (4, 149–152).

From a nutritional perspective, diets characterized by high fat intake and elevated levels of saturated fatty acids have been proposed to modulate ER stress in IBD, although the supporting mechanistic evidence is predominantly indirect and non-intestinal. SFAs can disrupt ER membrane phospholipid composition and activate the UPR independently of protein misfolding, thereby raising the hypothesis that they may exert a direct cytotoxic effect on intestinal epithelial cells. In an already compromised epithelial environment, such stressors could further sensitize the mucosal barrier, promoting PERK–ATF4–CHOP-mediated apoptosis and exacerbating barrier dysfunction (149, 153). High-fructose diets likewise engage the IRE1 branch in non-intestinal experimental models (111). It is important, however, to specify which IRE1 output is involved, because this branch is functionally bipolar (Section 3.2). The adaptive XBP1s programme is required for intestinal homeostasis, and it is the loss of this output—not its excess—that confers susceptibility to intestinal inflammation and constitutes a genetic risk factor for human IBD (34). The pro-inflammatory consequences of nutritionally induced IRE1 activation are therefore hypothesized to reflect sustained engagement of the kinase-dependent TRAF2–ASK1–JNK/NF-κB output and to hyperactivated RIDD, both of which operate independently of XBP1 splicing. Where XBP1s has been linked to lipogenesis and to metabolic inflammation, the evidence derives from hepatic and adipose tissue rather than from the intestinal epithelium, and the two settings should not be conflated. Combined exposure to high-fat and high-fructose diets produces a synergistic increase in ER stress markers and apoptotic responses in non-intestinal experimental models, supporting the hypothesis that, Western dietary patterns and increased IBD risk and severity (111). The available human dietary data are likewise non-intestinal and do not demonstrate mucosal UPR activation in IBD.

The pathogenic consequences of ER stress in IBD are further shaped by which UPR output predominates and by its downstream effects on immune responses and epithelial integrity. Evidence from experimental colitis and cell-culture studies indicates that sustained IRE1 signaling promotes Th1 and Th17 responses that are central to the pro-inflammatory immune profile of Crohn's disease (153); as set out in Section 2.2, this effect is mediated by the kinase-dependent TRAF2–ASK1 scaffold and the consequent activation of JNK and NF-κB rather than by XBP1 splicing. Because the corresponding human evidence is correlative, the proposition that nutritionally induced ER stress directly influences adaptive immune responses remains a hypothesis. In parallel, experimental evidence indicates that sustained IRE1 signaling promotes epithelial apoptosis and barrier disruption, increasing exposure to luminal antigens and perpetuating chronic inflammation (153). This distinction carries therapeutic weight: although this implication remains interpretive, an intervention that lowers total IRE1 activity would attenuate the injurious kinase output but would simultaneously compromise the protective XBP1s programme upon which the secretory epithelium depends.

Isoform specificity adds a further layer. Evidence from experimental colitis and microbial-colonization models shows that IRE1β (ERN2) is restricted to the mucosal epithelium and, within the intestine, is enriched in goblet cells, where it is induced by microbial colonization and supports MUC2 folding, goblet cell maturation, and assembly of the inner mucus layer (29, 30). Because IRE1β function is microbiota-dependent, these findings support the hypothesis that dietary patterns that alter microbial composition may modulate mucus barrier competence indirectly, in addition to any direct effect on epithelial secretion; loss of IRE1β function increases susceptibility to experimental colitis and therefore represents a plausible route by which diet-induced dysbiosis could weaken mucosal defense. It has been proposed that such mechanisms give rise to disease subtype-specific outcomes, with mucus barrier failure predominating in ulcerative colitis and transmural, autophagy-linked epithelial stress predominating in Crohn's disease. This proposal should be regarded as a hypothesis: it is consistent with the available isoform and genetic data, but it has not been tested by direct comparison of UPR branch activity between the two disease subtypes in matched human cohorts (154–156).

Conversely, certain dietary components may exert protective effects by attenuating ER stress. Indirect evidence from cell-culture and non-intestinal experimental models, together with limited intestinal cellular evidence, indicates that flavonoids, polyphenols, and specific fatty acids have been shown to reduce ER stress markers while enhancing autophagy and antioxidant defense mechanisms, thereby improving cellular stress adaptation in intestinal epithelial cells (107, 157, 158). Compounds such as quercetin and resveratrol lower GRP78, PERK, IRE1, and CHOP expression in both epithelial and immune cells, which supports the hypothesis that they may weaken the interaction between ER stress and pro-inflammatory signaling and could be particularly relevant in limiting chronic immune activation in IBD. The direction of this effect none the less requires careful interpretation. Reduced expression of GRP78 and of the three sensors in these experiments is most plausibly a consequence of a lowered upstream stress burden—that is, a readout of successful adaptation—rather than evidence that pharmacological silencing of the UPR is itself desirable. The distinction matters, because reduced mucosal GRP78 is itself a feature of Crohn's disease (2, 3, 9) and because each sensor mediates protective as well as injurious outputs (Section 2). Accordingly, and in the absence of direct human IBD evidence for this mechanism, nutritional strategies in this field are therefore better framed as reducing stress input and preserving adaptive capacity than as suppressing UPR signaling *per se* (159).

The interaction between nutrition and ER stress may also be mediated, at least in part, by the gut microbiota. Experimental evidence from high-fat-diet-fed obese mice indicates that certain flavonoids, such as rutin, have been shown to improve microbial composition while reducing lipopolysaccharide levels and pro-inflammatory cytokine production, accompanied by suppression of ER stress markers (107). Because these findings derive from an obesity model rather than experimental colitis, they provide only indirect support for IBD and suggest the hypothesis that nutritional effects on ER function may occur through both direct cellular and indirect microbial mechanisms (160).

Micronutrients also contribute to ER homeostasis. Indirect evidence from HepG2 cells indicates that riboflavin deficiency impairs protein folding processes, leading to the accumulation of misfolded proteins and activation of ER stress responses (117). Increased CHOP expression and enhanced apoptosis have also been observed in this model; however, their proposed consequences for epithelial barrier resilience and mucosal susceptibility in IBD remain hypothetical because they have not been demonstrated in intestinal tissue. The available human IBD evidence derives from an uncontrolled riboflavin intervention study that did not measure mucosal UPR activity and therefore does not establish this mechanism.

As an interpretive synthesis of the evidence distribution summarized in Table 1, these findings are consistent with ER stress functioning as one mechanistic pathway through which nutrition may influence intestinal inflammation, although the strength of the supporting evidence differs markedly between the individual steps of this argument (Table 1). Dietary patterns and nutrient composition demonstrably modulate ER stress in experimental systems, and ER stress in turn influences epithelial barrier integrity and immune responses; the step that has not been demonstrated is that dietary modulation of mucosal ER stress occurs in patients. While ER stress-inducing dietary factors may plausibly contribute to disease onset and progression, and while nutritional strategies that support cellular adaptation may offer complementary approaches, both propositions remain to be tested directly. This perspective nevertheless supports considering nutrition not only as a symptomatic intervention but as a potential modulator of underlying pathophysiological processes.

## 5 Clinical and translational evidence in humans

A recurrent criticism of the ER stress literature in IBD is that it rests disproportionately upon rodent models and immortalized cell lines. That criticism is largely fair, but it is not the whole picture: a body of human evidence has accumulated over the past 15 years, derived from mucosal biopsies, surgical specimens, patient-derived organoids, and genotype-stratified cohorts. Table 3 assembles this evidence in a single place, specifying for each study the sample examined, the ER stress markers measured, and the principal finding. Presenting the human data explicitly serves two purposes: it establishes what can legitimately be claimed at present, and it makes visible the specific design features that the field still lacks.

**Table 3:** Human and patient-derived studies of ER stress in inflammatory bowel disease.

References	Sample / population	ER stress markers assessed	Principal findings	Evidence level
Shkoda et al. (170)	Primary IEC from patients with IBD; IL-10-deficient mice	GRP78, ATF6	GRP78 induction in inflamed epithelium; IL-10 blocks ATF6 recruitment to the GRP78 promoter	Direct human IBD evidence (primary IEC) + experimental colitis (IL-10 knockout)
Kaser et al. (34)	Ileal and colonic tissue; genetic association cohorts	XBP1s/XBP1u, GRP78	Hypomorphic XBP1 variants confer risk for both CD and UC; epithelial UPR abnormality	Direct human IBD evidence (tissue + genetic association)
Bogaert et al. (171)	Colonic and ileal biopsies; 23 controls, 15 UC, 54 CD	HSPA5/GRP78, PDIA4, XBP1s	Markers increased in colonic IBD at mRNA and protein level; ATF6 and IRE1 branches activated; colonic–ileal difference	Direct human IBD evidence (biopsy)
Tréton et al. (161)	Uninflamed (quiescent) colonic mucosa, UC vs. controls	GRP78, p-eIF2α, XBP1s, translational readouts	ER stress and altered translation present in the absence of active inflammation	Direct human IBD evidence (biopsy, quiescent disease)
Deuring et al. (65)	Ileal biopsies, quiescent CD, stratified by ATG16L1 genotype	GRP78, p-eIF2α (Paneth cell IHC)	Risk allele-restricted Paneth cell ER stress without active disease; correlates with bacterial persistence	Direct human IBD evidence (genotype-stratified biopsy)
Dotti et al. (167)	Colonic organoids from UC patients and controls	Epithelial transcriptional programmes	Alterations of the epithelial stem cell compartment persist *ex vivo*, independent of the inflammatory milieu	Intestinal cellular evidence (patient-derived organoids)
Hanaoka et al. (48)	UC-associated and sporadic colorectal dysplasia and carcinoma	ATF6 (IHC)	ATF6 marks pre-cancerous atypical change; distinguishes low-grade dysplasia from regenerative epithelium better than p53	Direct human evidence (UC-associated dysplasia specimens)
Coope et al. (172)	Surgical specimens, CD; mucosa vs. mesenteric adipose tissue	GRP78, p-PERK, p-eIF2α, CHOP	ER stress activated in intestinal mucosa but not in mesenteric adipose tissue; associated with inflammation	Direct human IBD evidence (surgical specimens)
Grootjans et al. (173)	IEC-specific ER stress model (mouse) with human relevance	XBP1, IgA response	Epithelial ER stress induces a protective polyreactive IgA response — a beneficial consequence of UPR activation	Experimental colitis (IEC-specific mouse model)
Rees et al. (2)	Enteroids derived from patients with IBD	Epithelial stress and defense responses	Patient-derived enteroids display dysregulated epithelial responses retained in culture	Intestinal cellular evidence (patient-derived enteroids)
Khaloian et al. (67)	Ileal tissue, CD; postoperative cohort	Mitochondrial function, Paneth cell markers	Mitochondrial impairment drives stem cell transition to dysfunctional Paneth cells and predicts postoperative recurrence	Direct human IBD evidence (postoperative cohort) + experimental
Stengel et al. (43)	Ileal IEC from patients with CD; patient-derived and murine organoids	Activated (cleaved) ATF6, HSPA5, CSNK2B	Activated ATF6 elevated in CD ileal IEC; drives TNF and other cytokines upon ER stress	Direct human IBD evidence (ileal IEC) + intestinal cellular (organoids)
Solà Tapias et al. (174)	Human colonic tissue and organoids, UC	ER stress markers; trypsin activity	ER stress linked to increased trypsin activity affecting epithelial function	Direct human IBD evidence (tissue + organoids)
Al-Shaibi et al. (56)	Patients with biallelic AGR2 loss-of-function variants	Mucus barrier integrity, UPR activation	Monogenic infantile-onset IBD caused by failure of a single ER folding factor	Direct human evidence (monogenic IBD)
Rodrigues et al. (24)	Colonic mucosa, UC vs. controls	PERK, IRE1, GRP78, CHOP	Activation of both the PERK and the IRE1 branch in UC mucosa	Direct human IBD evidence (biopsy)
Wu et al. (25)	Intestinal tissue from patients with IBD	GRP78, CHOP and related markers; TLR2	ER stress markers upregulated; contribution to TLR2 pathway-mediated inflammatory responses	Direct human IBD evidence (tissue)
Cao et al. (90)	Inflamed tissue from patients with IBD; sorted ILC3s	IRE1α/XBP1	IRE1α/XBP1 activated in ILC3s from patients; sustains cytokine output; circadian expression pattern in mice	Direct human IBD evidence (sorted ILC3) + experimental colitis
Sharma et al. (59)	UC patient cohort; human macrophages	ORMDL3, IL-1β, ER–mitochondria contacts	ORMDL3 expression tracks disease activity and IL-1β; regulates NLRP3 inflammasome activation	Direct human IBD evidence (patient cohort) + experimental colitis + cell culture

ATF6f, cleaved ATF6; CD, Crohn's disease; IEC, intestinal epithelial cell; IHC, immunohistochemistry; UC, ulcerative colitis; UPR, unfolded protein response.

Two findings in Table 3 deserve particular emphasis, because together they inform, although they do not resolve, the temporality question raised in the concluding section. First, direct human IBD evidence shows that ER stress and altered translational control are demonstrable in uninflamed, quiescent colonic mucosa from patients with ulcerative colitis, that is, in the absence of active inflammation (161). Second, genotype-stratified direct human IBD evidence shows that patients with quiescent Crohn's disease who carry the ATG16L1 risk allele show Paneth cell ER stress restricted to that genotype, again without active disease (65). These observations argue against a simple model in which ER stress occurs only as a downstream consequence of active mucosal inflammation and together provide strong human evidence that proteostatic dysfunction can persist independently of clinical disease activity. However, they do not establish that ER stress precedes disease onset. A third observation cuts in the opposite direction and should be reported with equal prominence: experimental evidence, supported by observations in healthy humans with defective autophagy, indicates that epithelial ER stress drives a protective, polyreactive IgA response that limits enteric inflammation (158). This is consistent with the adaptive–maladaptive framework of Section 2.4 and is a further reason to resist describing ER stress as uniformly deleterious (162).

The limitations of this evidence base are equally clear from the table. Almost every human study is cross-sectional; sample sizes are modest; the markers measured differ between studies, so that results cannot be pooled; whole-mucosa homogenates dominate, obscuring the cell type-specific effects established in Section 4; and, critically for the present review, not one of these studies incorporated a controlled nutritional exposure. The nutrition–ER stress axis has therefore never been tested directly in human intestinal tissue. Every statement connecting a specific dietary component to mucosal UPR activation in patients remains an inference drawn from parallel literatures.

### 5.1 ER stress biomarkers: current status and requirements for clinical use

The prospect of using UPR components as clinical biomarkers is frequently raised, and the concluding section of this review endorses it as a research direction. It is nevertheless important to state where the field currently stands. Table 4 lists the seven markers most often proposed, together with the sample in which each is measured, the clinical role for which it might plausibly be developed, and the level of evidence presently supporting it, expressed as the type of study in which it has been evaluated.

**Table 4:** Candidate ER stress biomarkers in inflammatory bowel disease.

Marker	Sample	Potential clinical role and principal limitation	Evidence level
GRP78 / BiP	Mucosal biopsy; isolated IEC (immunoblot, IHC, mRNA)	Global indicator of ER stress burden; monitoring. Limitation: direction of change is inconsistent — reduced in Crohn's disease mucosa but increased in inflamed colonic IBD, so it cannot be interpreted without knowing disease location and phase	Human studies (biopsy, cross-sectional) + animal models + cell culture
XBP1s and XBP1s/XBP1u ratio	Mucosal biopsy; isolated IEC; organoids	Susceptibility and mechanistic stratification, particularly in combination with XBP1 genotype. Limitation: direction of effect is compartment-dependent — adaptive in epithelium, pro-inflammatory in myeloid and innate lymphoid cells	Human studies (biopsy + genetic association) + animal models + cell culture
p-PERK	Mucosal biopsy (immunoblot, IHC)	Branch-specific readout of PERK activation. Limitation: technically demanding, antibody-dependent, and not yet compared across disease subtypes in matched cohorts	Human studies (UC mucosa, surgical specimens) + animal models + cell culture
p-eIF2α	Mucosal biopsy; Paneth cell IHC	Genotype-restricted marker of secretory cell stress, demonstrated in ATG16L1 risk allele carriers. Limitation: shared node of the ISR — can be raised by GCN2, PKR or HRI without ER protein misfolding, so it is not PERK-specific without ISR controls	Human studies (genotype-stratified CD) + animal models + cell culture
ATF4	Mucosal biopsy; isolated IEC	Indicator of adaptive ISR output upstream of CHOP. Limitation: human intestinal data are sparse; almost all evidence is preclinical	Animal models + cell culture; limited human data
CHOP (DDIT3)	Mucosal biopsy; isolated IEC	Indicator of terminal, maladaptive UPR and of the adaptive-to-apoptotic transition; the most mechanistically interpretable marker in the table. Limitation: no established threshold separating adaptive from terminal signaling in human tissue	Human studies (biopsy) + animal models (causal — CHOP-deficient mice protected from colitis) + cell culture
Cleaved ATF6 (ATF6f / nATF6)	Ileal IEC; patient-derived organoids	Crohn's disease-specific marker of epithelial ATF6 activation; candidate for stratification. Limitation: detection of the cleaved fragment is technically difficult; no ATF6 loss-of-function human or IEC-specific data exist to establish necessity	Human studies (CD ileal IEC, organoids) + animal models (transgenic nATF6) + cell culture

The evidence-level column states the type of study in which each marker has been evaluated. None of these markers has undergone analytical validation, none has an established cut-off value, and none has been assessed prospectively against a clinical endpoint; all should therefore be regarded as exploratory research markers. IEC, intestinal epithelial cell; IHC, immunohistochemistry; ISR, integrated stress response.

A methodological appraisal across all evidence levels represented in Table 4 identifies three limitations that are more consequential than any marker-specific consideration. The first is directional inconsistency. GRP78 illustrates the problem: it is reported as reduced in the intestinal mucosa of patients with Crohn's disease yet increased in inflamed colonic IBD tissue, and it falls in response to protective dietary compounds. A marker whose direction of change depends on disease location, disease phase and cell type cannot serve as a monitoring biomarker until those dependencies are mapped. The second is specificity. Phosphorylated eIF2α is the most widely used readout of PERK activity, but as set out in Section 2.1 it is the shared node of the integrated stress response and can be raised by GCN2, PKR or HRI without any ER protein misfolding; interpreting it as an ER stress marker without ISR-specific controls is not defensible. The third is accessibility: every marker in Table 4 requires mucosal tissue, and no validated circulating or fecal surrogate of intestinal UPR activity exists.

Consequently, none of these markers has undergone analytical validation, none has an established cut-off value, and none has been evaluated prospectively against a clinical endpoint. They should be described as exploratory research markers rather than as candidate clinical biomarkers, and the appropriate next step is not clinical implementation but the standardization of measurement—agreement on which markers, in which compartment, at which cell-type resolution, and in inflamed vs. uninflamed tissue sampled in parallel.

## 6 Future research directions

Future research should prioritize human-based studies to better define the role of ER stress in IBD pathogenesis. Investigations incorporating ER stress biomarkers in intestinal biopsy samples, as well as clinical trials examining the combined effects of dietary interventions and ER stress modulation, are likely to provide valuable insights. Longitudinal cohort studies may further clarify the temporal relationship between nutrition, ER stress, and disease progression. In addition, a deeper understanding of how ER stress influences intestinal barrier integrity may open new avenues for early diagnosis and intervention. Targeting ER stress also represents a promising therapeutic direction. Strategies aimed at modulating UPR signaling or enhancing cellular adaptive capacity may help reduce inflammation and preserve epithelial function (163).

As a research priority, controlled dietary interventions with mechanistic endpoints represent the most direct route to testing the axis proposed in this review. Direct human IBD evidence establishes the feasibility of rigorous dietary trials: an ordinary food-based diet replicating exclusive enteral nutrition alters the microbiome and induces clinical response (164); the Crohn's disease exclusion diet with partial enteral nutrition induces sustained remission in a randomized trial (165); and the specific carbohydrate and Mediterranean diets produce comparable symptomatic remission (166). None of these trials, however, incorporated a mucosal mechanistic endpoint; they therefore provide evidence for clinical feasibility but not for the proposed nutrition–ER stress mechanism. Embedding paired pre- and post-intervention biopsies with predefined UPR readouts into future trials of this design would provide a direct test of that hypothesis.

As a methodological research priority, patient biopsy studies require standardization before they can be pooled. As Table 3 shows, existing studies measure different markers in different compartments at different disease phases, which currently precludes meaningful quantitative synthesis. Four design features should become standard: parallel sampling of inflamed and uninflamed mucosa from the same patient, since the quiescent-tissue findings of Tréton et al. (161) and Deuring et al. (65) indicate that the uninflamed compartment provides distinct information that would be missed by sampling inflamed tissue alone; a defined marker panel spanning all three branches rather than a single readout; cell type-resolved measurement, given the lineage-specific effects established in Section 4; and genotype stratification, at minimum for XBP1, ATG16L1, and NOD2.

As a research priority building on existing intestinal cellular evidence, patient-derived organoids offer experimental control that human tissue studies cannot provide. Existing intestinal cellular evidence indicates that organoids retain donor-specific epithelial phenotypes *ex vivo* (2, 167), which permits controlled exposure to fatty acids, bile acids, short-chain fatty acids, and polyphenols at defined concentrations, with the albumin-to-fatty-acid ratio reported, and with genuine dose–response designs of the kind whose absence was noted in Section 5. Genotype-stratified organoid biobanks would additionally allow the gene–environment interactions described in Section 4 to be tested directly, by exposing organoids of defined XBP1, ATG16L1, or NOD2 genotype to identical nutritional stressors.

As a research priority building on existing microbial-colonization and germ-free evidence, Germ-free and gnotobiotic models are required to separate direct nutrient effects on the epithelium from effects mediated by the microbiota. The precedent is well established: Ern2 expression is induced only after microbial colonization (30), and tumorigenesis in nATF6-transgenic mice is entirely prevented by germ-free housing (44), although the latter is a tumorigenesis model rather than an experimental colitis model. Given that four of the mechanisms described in Section 6.3 are microbially mediated, no dietary intervention study in conventional animals can attribute an effect to a nutrient rather than to its microbial metabolites without this control (168).

As a research priority addressing the data gaps identified in Sections 4 and 4.1, cell type-specific conditional deletion represents a direct experimental approach. No conditional deletion of a UPR sensor has been reported in tuft cells, enteroendocrine cells, neutrophils or endothelium, and appropriate driver lines are available for targeting these compartments. Crossing such drivers to floxed Ern1, Eif2ak3, or Atf6 alleles would establish whether the UPR is functionally required in these compartments, or whether their omission from the present literature reflects genuine dispensability rather than absence of investigation. This is a tractable programme of work rather than a speculative one.

Time-series and longitudinal designs address what is arguably the central conceptual weakness of the field. The entire adaptive-vs.-maladaptive framework developed in Section 2.4 is defined by the duration and amplitude of signaling, yet almost all available data—human and experimental alike—consist of single time points. The circadian rhythmicity of IRE1α/XBP1 expression in intestinal ILC3s (90) indicates that even the time of day at which a biopsy is taken may influence the result. Longitudinal sampling across disease phases, measurement of autophagic flux rather than static LC3-II and p62 levels as discussed in Section 3.1, and prospective designs relating early UPR activation to subsequent clinical outcome are all required before the temporal claims made throughout this literature can be regarded as tested.

As a therapeutic research priority, development in this area should be directionally informed rather than sensor-directed. As argued in Sections 2.4 and 4.1, each UPR branch mediates both protective and injurious outputs, and the direction of effect is compartment-dependent; agents that suppress a sensor globally would be expected to impair epithelial adaptation while attenuating immune activation. Strategies that preserve adaptive output while limiting terminal signaling—selective suppression of the CHOP arm, restoration of autophagic flux in ATG16L1 risk allele carriers, or support of ERAD capacity—are conceptually preferable. However, these strategies have not been validated clinically, and the available proof of principle is limited to experimental colitis, in which chemical chaperones attenuate disease (68). Nutritional strategies belong in the same framework: their objective should be to reduce the stress input and preserve adaptive reserve rather than to suppress UPR signaling as such.

## 7 Conclusion

As a synthesis of the evidence reviewed, direct human IBD studies support the involvement of the UPR in intestinal inflammation, whereas current evidence suggests that ER stress may represent an important mechanistic pathway linking nutrition and intestinal inflammation; however, direct causal evidence for this nutritional link in humans remains limited. This formulation is deliberately more restrained than that commonly encountered in this literature, and it follows directly from the evidence classification applied throughout this review. Indirect evidence from experimental systems indicates that dietary patterns and nutrient composition modulate UPR signaling in experimental systems: saturated fatty acids, cholesterol, and high fructose intake activate PERK- and IRE1-dependent outputs, whereas polyunsaturated fatty acids, polyphenols, and certain micronutrients attenuate them. What remains unproven is that these effects occur in the intestinal mucosa of patients, at intakes attainable through diet, and with consequences for the course of disease.

A methodological appraisal of the evidence distribution presented in Table 1 makes the structure of this evidence base explicit, and the pattern it reveals should be stated plainly. Almost the entire mechanistic case for nutritional modulation of ER stress rests on indirect evidence—hepatocytes, adipocytes, macrophages, neuronal and osteoblast lines, and rodent models of obesity and insulin resistance. Only one of the compounds reviewed has been examined in an intestinal epithelial cell line, and none has been examined in primary intestinal epithelium or in patient-derived organoids with a UPR readout. Conversely, direct human IBD evidence is limited to clinical studies of curcumin, resveratrol, riboflavin, and omega-3 fatty acids, whereas the berberine trial concerned colorectal adenoma recurrence rather than IBD. The mechanistic and the clinical literatures therefore do not overlap at any point. Accordingly, the nutrition–ER stress–IBD axis remains a coherent hypothesis supported by separate mechanistic and clinical literatures but untested at their proposed point of connection.

Three propositions are nevertheless supported by direct human evidence and should not be diluted by the caution expressed above. First, UPR components contribute to genetic susceptibility to IBD: hypomorphic XBP1 variants confer genetic risk for both Crohn's disease and ulcerative colitis, and biallelic AGR2 loss of function causes a monogenic form of infantile-onset IBD. Second, ER stress can occur independently of active clinical inflammation, since it is demonstrable in uninflamed colonic mucosa in ulcerative colitis and in Paneth cells of quiescent Crohn's disease restricted to carriers of the ATG16L1 risk allele. These findings do not, however, establish that ER stress precedes disease onset. Third, direct human IBD evidence shows that activated ATF6 is elevated in the ileal epithelium of patients with Crohn's disease, whereas its capacity to drive cytokine expression is supported by intestinal cellular evidence. These findings concern the UPR itself rather than its nutritional modulation, and their evidential weight should not be transferred to the dietary claims.

The dual role of the UPR remains the principal conceptual obstacle to translation. Asan established molecular principle, supported in the IBD context by direct human and experimental colitis evidence, each branch mediates both protective and injurious outputs, and the direction of effect depends upon the duration and amplitude of signaling and upon the cell type involved, as set out in Sections 2, 4, and 4.1 and summarized in Figure 1. XBP1s is protective in the epithelium but pro-inflammatory in macrophages, dendritic cells, and innate lymphoid cells; epithelial ER stress also drives a protective IgA response. Neither ER stress in general nor any individual sensor can therefore be treated as a therapeutic target in the singular, and the direction in which an intervention moves a particular output matter more than whether it raises or lowers a marker.

As a research priority that is not yet supported by direct human evidence, the practical implication for nutrition science is a change of objective rather than of ambition. Rather than seeking dietary compounds that suppress UPR markers, the more defensible aim is to reduce the proteostatic load carried by the intestinal epithelium and to preserve adaptive reserve: potentially through fiber intake sufficient to sustain butyrate production and mucus turnover, through limitation of the saturated fat and fructose exposures for which experimental evidence of ER stress induction is most consistent, and through correction of micronutrient deficiencies that impair oxidative protein folding. Whether such an approach alters the course of IBD is not currently known. It becomes answerable as soon as mucosal ER stress endpoints are incorporated into the dietary intervention trials that this field has already shown itself capable of conducting—and that step, rather than further mechanistic work in non-intestinal models, is what the nutrition–ER stress–IBD hypothesis now requires.
